# Global, regional, and national burden of ovarian cancer attributable to high body mass index, 1990–2021: insights from the global burden of disease study 2021

**DOI:** 10.3389/fonc.2025.1568716

**Published:** 2025-10-31

**Authors:** Yinghua Wang, Yuexin Yu

**Affiliations:** Center of Reproductive Medicine, General Hospital of Northern Theater Command, Shenyang, China

**Keywords:** ovarian cancer, high body mass index, global burden of disease, BAPC, obesity

## Abstract

**Objective:**

Building on established evidence linking excess body fatness to increased risk of ovarian cancer, this research aims to assess the global and regional disease burden of ovarian cancer attributable to high body mass index (BMI) from 1990 to 2021 using data from the Global Burden of Disease (GBD) study. Here, we specifically focus on quantifying trends in age-standardized mortality rates (ASMR) and age-standardized disability-adjusted life year rates (ASDR) to elucidate evolving epidemiological patterns and regional disparities.

**Methods:**

Comprehensive data were extracted from the GBD 2021 database to analyze trends in ASMR, ASDR, absolute deaths, and disability-adjusted life years (DALYs) attributable to high BMI-related ovarian cancer from 1990 to 2021, stratified by global, regional, and socio-demographic index (SDI) quintiles. The key methodological approaches included Joinpoint regression analysis to identify significant temporal changes in ASMR and ASDR trends and correlation analysis to determine associations between disease burden (ASMR/ASDR) and SDI. Additionally, future projections for ASMR and ASDR burdens from 2022 to 2050 were generated using a Bayesian Age-Period-Cohort (BAPC) framework, accounting for demographic shifts and inherent uncertainties.

**Results:**

Between 1990 and 2021, global ASMR and ASDR for ovarian cancer attributable to high BMI remained relatively stable. However, the absolute number of deaths and DALYs increased substantially over this period. ASMR and ASDR exhibited a strong positive correlation with SDI, with the highest burden observed in regions with SDI values between 0.7 and 0.8. While high-SDI regions experienced the greatest burden in both 1990 and 2021, an overall declining trend was observed. Conversely, regions across other SDI quintiles exhibited an increasing burden. BAPC projections suggest a continued global increase in the ovarian cancer burden attributable to high BMI, with global ASMR projected to reach 1.64 (95% uncertainty level (UI): 1.35–1.93) and ASDR to reach 54.63 (95% UI: 45.41–63.84) by 2050.

**Conclusion:**

This study highlights persistent and significant regional disparities in the ovarian cancer burden attributable to high BMI, strongly associated with SDI, and projects a concerning increase in the future global burden. Our collective findings underscore the urgent need for targeted and proactive interventions to mitigate the impact of high BMI on ovarian cancer outcomes worldwide.

## Introduction

Ovarian cancer is a highly lethal malignancy of the female reproductive system, with ~240,000 new cases annually and a five-year survival rate below 45% (1, 2). This poor prognosis is primarily due to frequent late-stage diagnosis and therapeutic challenges, evidenced by the status of ovarian cancer as the leading cause of gynecologic cancer mortality in the United States (3, 4). Common causes include genetic mutations, hormonal influences such as prolonged ovulation, and lifestyle factors. Among multiple lifestyle factors, excess body fatness has emerged as a significant risk factor for ovarian cancer.

Obesity, defined as high body mass index (BMI ≥30 kg/m²), is a well-established public health threat that contributes substantially to the global disease burden (5). In the U.S., obesity is implicated in approximately 40% of cancer diagnoses, with robust epidemiological evidence linking obesity to thirteen malignancies, including ovarian cancer (6). Mechanistic studies have demonstrated that elevated excess body fatness promotes the progression of epithelial ovarian cancer through modulation of transforming growth factor alpha (7). Concurrently, estrogen signaling plays a critical role in regulating key malignant behaviors in ovarian cancer, including cellular proliferation, invasion, and epithelial-mesenchymal transition. Postmenopausal weight gain is strongly linked to increased circulating estrogen levels (8) and altered estrogen metabolism patterns (9), both of which potentially contribute to elevated risk of ovarian cancer. However, comprehensive global data quantifying the specific disease burden of ovarian cancer attributable to excess body fatness, particularly in terms of mortality and incidence, remain limited.

The Global Burden of Disease (GBD) study provides a robust, internationally recognized platform for assessing the global impact of diseases and associated risk factors (10). Against the backdrop of escalating global obesity, projected to affect 3.8 billion adults by 2050 (11), the burden of ovarian cancer is expected to increase simultaneously, with forecasts indicating a rise in age-standardized mortality rates (ASMR) to 2.54 per 100,000 by 2030) (12) and continued growth in case numbers by 2050 (13). This dual growth trajectory underscores the expanding impact of excess body fatness on the global ovarian cancer burden. As the GBD framework describes obesity as BMI, this study aims to adopt high BMI as the primary metric of GBD data to: (1) estimate the global ASMR and ASDR of ovarian cancer attributable to high BMI, (2) analyze temporal trends in this burden between 1990 and 2021, and (3) characterize geographic variations across regions and nations. This evaluation is intended provide essential epidemiological evidence to underpin the formulation of evidence-based prevention and control policies for ovarian cancer attributable to high BMI.

## Methods

### Data source

Data for this study were sourced from the Global Burden of Disease (GBD) database, which currently provides health information spanning the period from 1990 to 2021. The objective of the GBD is to comprehensively assess the global impact of diseases, injuries, and risk factors on population health through integration of a wealth of information derived from epidemiological studies and population models across 204 countries and regions, thereby offering key indicators for evaluating health status. Through systematic analysis of these health metrics, the GBD serves as a critical resource for researchers and policymakers, enabling a deeper understanding of the patterns and trends in disease burden, in turn, facilitating more effective responses to public health challenges (14).

In this study, we utilized the Global Health Data Exchange GBD Results Tool (http://ghdx.healthdata.org/gbd-results-tool) to download data concerning ovarian cancer attributable to high BMI (population attributable fractions for BMI ≥30 kg/m²; GBD cause and risk factor codes). The dataset included critical indicators such as mortality rates and disability-adjusted life years (DALYs). Furthermore, demographic statistics were incorporated, including age, gender, and region, facilitating stratified analysis. Data were grouped into 5-year age intervals beginning at 20 years, sex-specific categories, and geographic divisions across 204 nations, 21 GBD regions, and 5 sociodemographic index (SDI) tiers. The SDI, a composite measure of socioeconomic development, ranges from 0 (lowest development) to 1 (highest development). The Population Attributable Fraction (PAF) quantifies the impact of a risk factor on a given outcome, representing the proportion of cases in the population that could be prevented if the risk factor were eliminated. Its calculation incorporates the risk function and the distribution of exposure across different age, sex, region, and year strata, and is conducted independently for each risk factor.

### Statistical analysis

Based on the GBD 2021 database, we conducted a comprehensive analysis of the mortality and DALY statistics associated with ovarian cancer attributable to high BMI. Age-standardized rates (ASR) per 100,000 population were computed using the direct standardization method. This process involves calculating weighted averages of age-specific rates, where each rate is multiplied by its corresponding age-group proportion in the WHO reference standard population. The weighted rates were summed and then adjusted to a 100,000-person denominator to yield final ASR estimates. ASMR and ASDR were calculated by comparing mortality and DALY data with the WHO reference standard population distribution for the years 2000–2025. During data processing, to ensure the reliability of the results, 95% uncertainty intervals (UI) were calculated for both ASMR and ASDR. The 95% UIs were derived from 1000 draws of the posterior distribution, with the 2.5^th^ and 97.5^th^ percentiles representing the lower and upper bounds, respectively. Through the Joinpoint regression model, we calculated the annual percentage change (APC) for each trend segment and the average annual percentage change (AAPC) over the entire study duration (1990–2021), with corresponding 95% confidence intervals and *P* values. The AAPC was computed as a weighted average of the APCs, with weights reflecting the length of each segment. A maximum limit of five Joinpoints was specified in the model to capture potential shifts in trends following the methodological framework proposed by Kim et al. (15). Additionally, we explored the distribution and evolving trends of ovarian cancer burden across different regions attributable to high BMI. To evaluate the relationship between the SDI and both ASMR and ASDR, Spearman correlation analysis was conducted. We utilized the Bayesian-Age-Period-Cohort (BAPC) package in R to model and forecast age-specific prevalence rates of ovarian cancer attributable to BMI from 1990 to 2021, with projections extending to 2050. The model adopts a Bayesian hierarchical framework and estimates parameters via the Integrated Nested Laplace Approximation (INLA) method. The detailed model specifications are shown in Supplementary Materials. All assumptions and model specifications were guided by the research of Riebler and Held on the BAPC model (16). Statistical analyses were conducted using R (version 4.4.1) and Joinpoint software (version 4.9.1.0).

## Results

### Age-related patterns of ovarian cancer mortality and DALY attributable to high BMI

**Figure 1:**
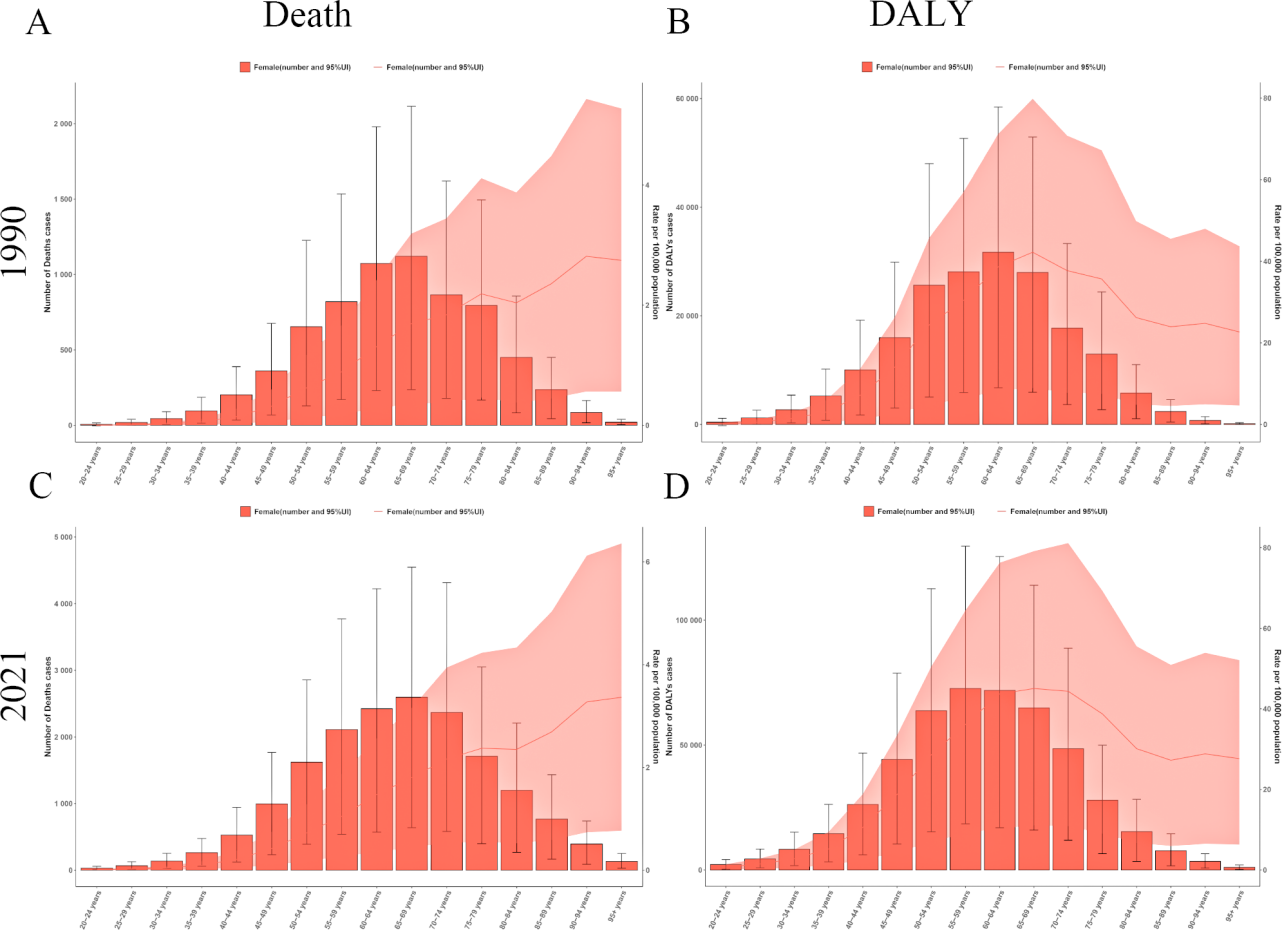
Age pattern of ovarian cancer burden attributable to high body-mass index in 1990 and 2021. **(A)** The death of ovarian cancer attributable to high body-mass index in 1990. **(B)** The DALY of ovarian cancer attributable to high body-mass index in 1990. **(C)** The death of ovarian cancer attributable to high body-mass index in 2021. **(D)** The DALY of ovarian cancer attributable to high body-mass index in 2021. DALY, disability-adjusted life years.

Statistics from 1990 and 2021 indicate an overall increasing trend in the mortality rate from ovarian cancer attributable to high BMI. With advancing age, the number of ovarian cancer deaths initially increased, peaking in the 65- to 69-year age group, followed by a decline (**Figures 1A, C**). This finding highlights the significant influence of age on ovarian cancer mortality, particularly among middle-aged and older adult women.

A similar age-related phenomenon was observed in analysis of DALY counts, illustrating an initial increase followed by a decline. However, the peak age groups of these two indicators exhibited slight differences. In 1990, the highest concentration of DALY years occurred in the 60- to 64-year group (**Figure 1B**), while data from 2021 showed a shift in the peak to the 55- to 59-year group (**Figure 1D**). Ultimately, both the 1990 and 2021 datasets indicate that the DALY rate due to ovarian cancer attributable to high BMI reaches an inflection point in the 65–69 age demographic, underscoring age as a key determinant of variations in the disease burden of ovarian cancer attributable to high BMI.

### Temporal trends in ASMR and ASDR of ovarian cancer attributable to high BMI

**Figure 2:**
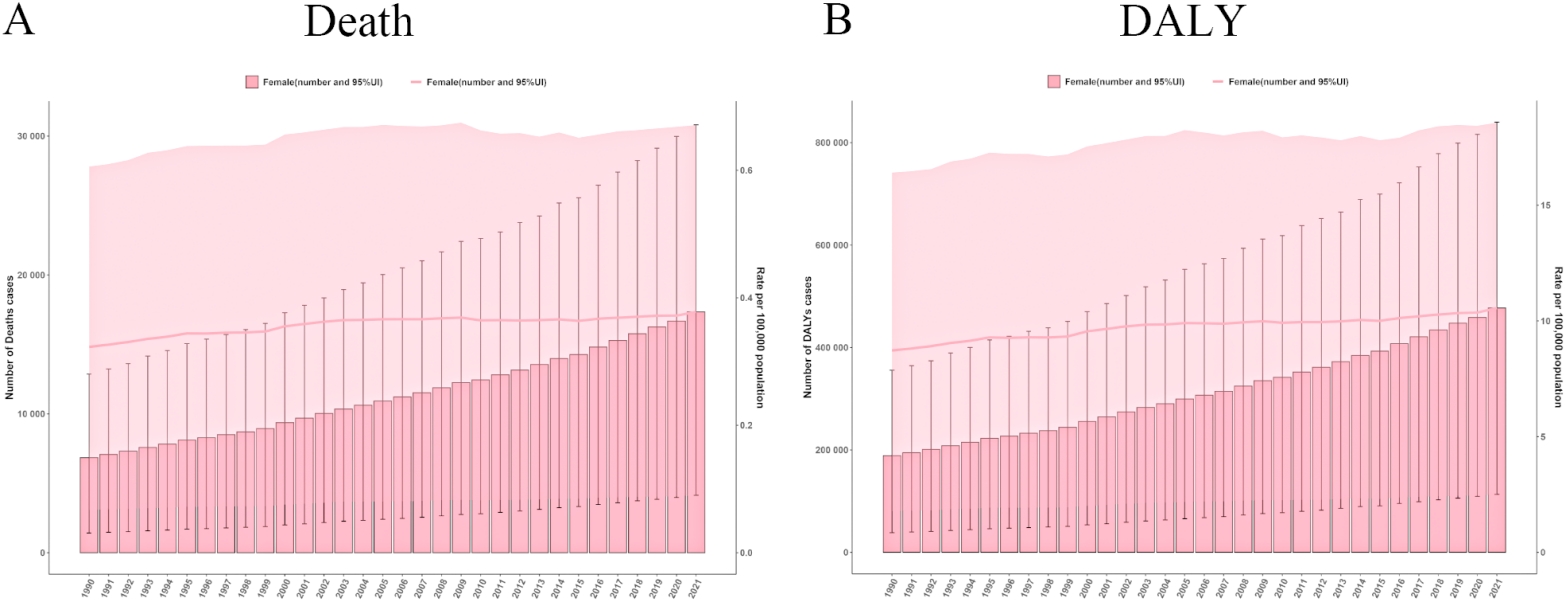
The global trends in ovarian cancer burden attributable to high body-mass index from 1990 to 2021. **(A)** Deaths number and age standardized mortality rate in ovarian cancer burden attributable to high body-mass index. **(B)** DALYs number and age-standardised DALYs rate in ovarian cancer burden attributable to high body-mass index. DALY, disability-adjusted life years.

From 1990 to 2021, the ASMR and ASDR for ovarian cancer attributable to high BMI showed minimal fluctuations. However, both the mortality rate and disease burden (DALY years) increased markedly over this period (**Figure 2**). Specifically, the number of ovarian cancer-related deaths attributable to high BMI escalated from 6,850 in 1990 (95% UI: 1,423–12,865) to 17,344 in 2021 (95% UI: 4,141–30,810). Similarly, the total DALY years increased from 188,874 in 1990 (95% UI: 38,401–355,691) to 477,248 in 2021 (95% UI: 113,449–840,002), highlighting substantial exacerbation of the disease burden over time (**Supplementary Table S1**).

The ASMR values fluctuated modestly between 0.32 and 0.38, indicating a relatively stable trend. Joinpoint regression analysis further illuminated temporal variations in ASMR trends (**Supplementary Figure S1**). The most pronounced upward trend in ASMR was observed from 1990 to 1993, with an annual percentage change (APC) of 1.28 (*P* < 0.05). In contrast, a significant decline was observed between 2008 and 2015, with an APC of -0.13 (*P* < 0.05), potentially reflecting the influence of improvements in public health policies and enhanced awareness of obesity-related cancer risk.

Conversely, the ASDR increased from 8.72 in 1990 (95% UI: 1.78 - 16.41) to 10.56 in 2021 (95% UI: 2.50 - 18.57), reflecting a progressive impact of high BMI on the overall public health burden. The Joinpoint analysis revealed a general upward trend in ASDR from 1990 to 2021, with a notable peak from 1998 to 2002, where the APC reached 1.36 (*P* < 0.05), signifying a marked escalation in disease burden. In contrast, the slowest change was recorded between 1995 and 1998, during which the APC was only 0.02 (*P* < 0.05).

### Regional disparities in ASMR and ASDR of ovarian cancer attributable to high BMI

**Figure 3:**
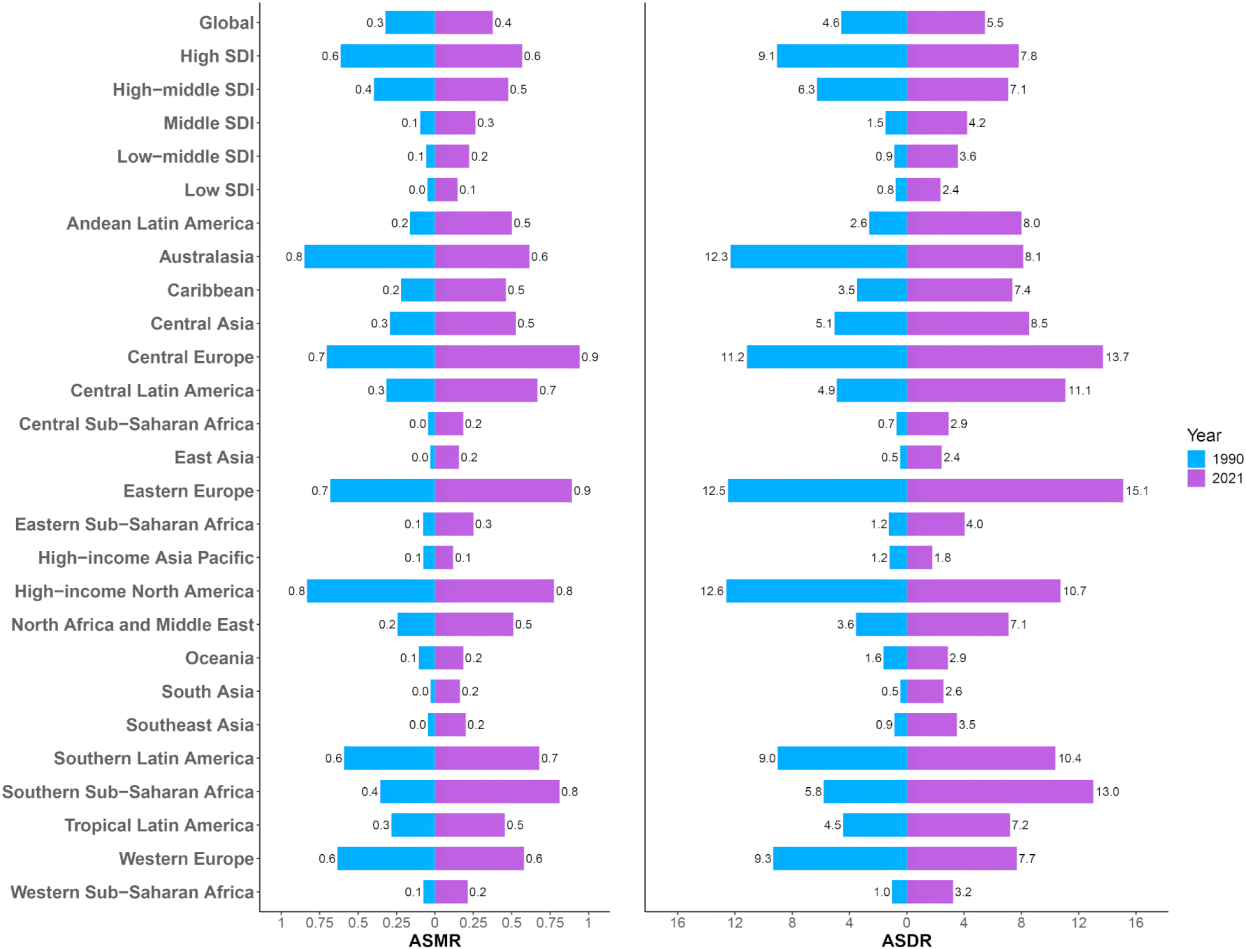
The ASMR and ASDR of ovarian cancer attributable to high body-mass index in 5 SDI and 21 GBD regions in 1990 and 2021. SDI, Socio-Demographic Index; GBD, Global Burden of Diseases; DALY, disability-adjusted life years. ASMR, age-standardized rates of mortality; ASDR, age-standardized rate of DALYs.

**Figure 4:**
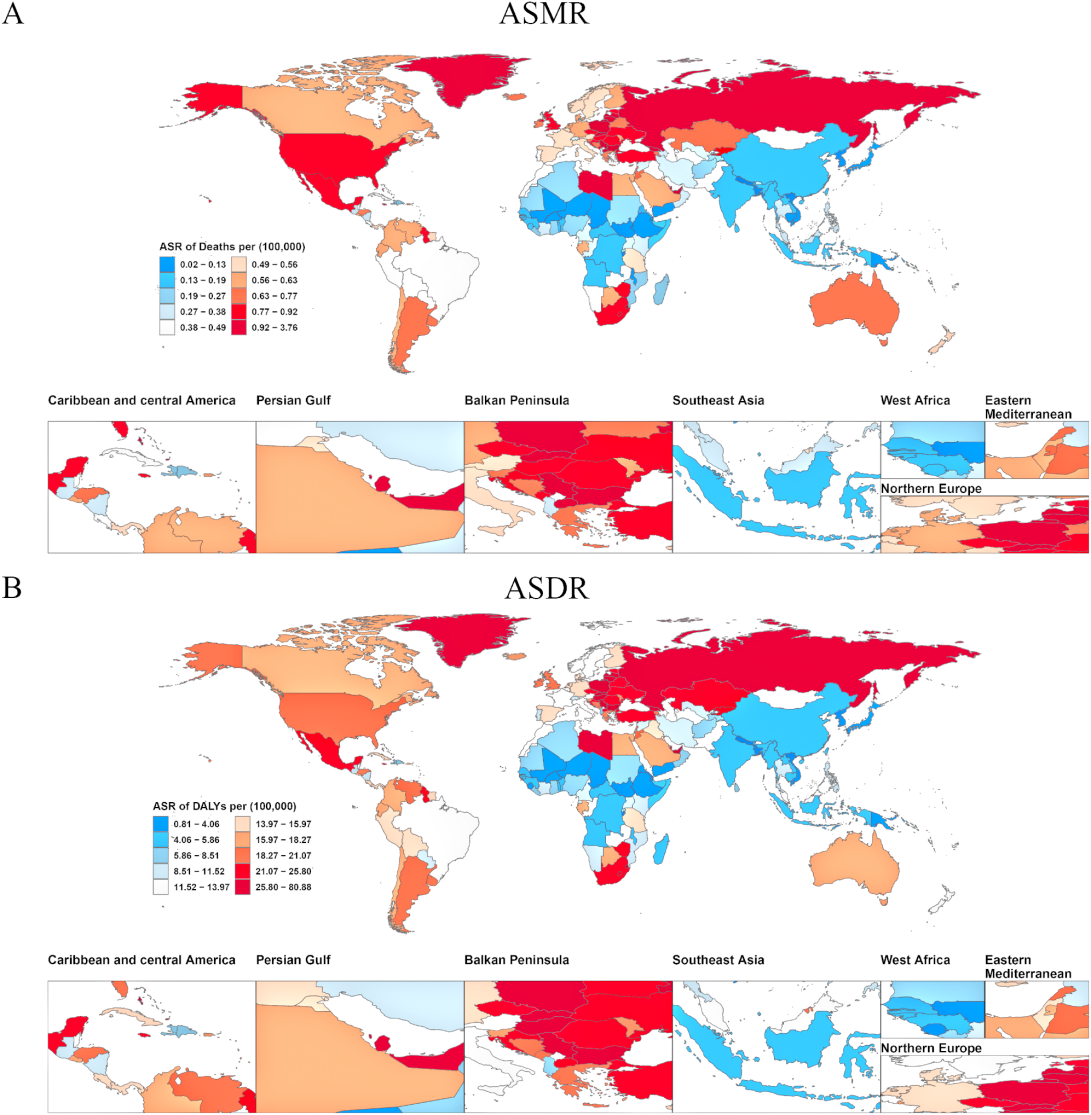
Age-standardized burden of ovarian cancer attributable to high body-mass index in 204 countries and territories in 2021. **(A)** World map of ASMR for ovarian cancer attributable to high body-mass index. **(B)** World map of ASDR for ovarian cancer attributable to high body-mass index. DALY, disability-adjusted life years; ASMR, age-standardized rates of mortality; ASDR, age-standardized rate of DALYs.

Analysis of ASMR data revealed significant regional disparities in ovarian cancer mortality attributable to high BMI across five SDI categories and 21 Global Burden of Disease GBD regions (**Figure 3**). High-SDI regions consistently exhibited the greatest ASMR burden. However, a notable decline was recorded in these regions from 1990 to 2021, with ASMR decreasing from 0.61 (95% UI: 0.13–1.15) to 0.57 (95% UI: 0.14–1.01), corresponding to an AAPC of −0.002 (95% UI: −0.002, −0.001) (**Supplementary Table S2**). In contrast, the other four SDI regions (middle-high, middle, low-middle, and low SDI) experienced an increase in ASMR over the same period, indicating a shifting global pattern in which the mortality burden is diminishing in high-resource regions while escalating elsewhere. Among the 21 GBD regions and 204 countries/territories, significantly higher ASMRs for ovarian cancer attributable to high BMI were reported in Australasia, High-income North America, Central Europe, Eastern Europe, Western Europe, and Southern Latin America, compared to other regions (**Figure 4**). Geographically, Europe emerged as the continent with the highest mortality burden overall. In 1990, Greenland had the highest recorded ASMR of 1.31 (95% UI: 0.32–2.47) (**Supplementary Table S3**). By 2021, the United Arab Emirates (UAE) had experienced the most dramatic increase in ASMR, rising from 0.83 (95% UI: 0.14–2.00) in 1990 to 3.73 (95% UI: 1.05–6.55), with an AAPC of 0.094 (95% UI: 0.088–0.101), thereby ranking as the region with the highest ASMR globally in 2021.

Analysis of ASDR aligned with the mortality findings while providing a comprehensive overview of health loss (**Figure 3**). Similar to ASMR, regions classified as high-SDI exhibited the highest overall disease burden in terms of ASDR, but displayed a significant downward trend from 1990 to 2021. The number of DALYs decreased from 16.78 (95% UI: 3.57–31.36) to 15.13 (95% UI: 3.79–26.82), corresponding to an AAPC of −0.057 (95% UI: −0.063, −0.051) (**Supplementary Table S2**). The ASDR in this region additionally declined during this period. Among the 21 GBD regions and 204 countries/territories, Australasia, High-income North America, Central Europe, Eastern Europe, Western Europe, and Southern Latin America consistently reported significantly elevated ASDR compared to other regions (**Figure 4**). In 1990, Greenland recorded the highest ASDR (37.54; 95% UI: 9.06–70.32) (**Supplementary Table S3**). By 2021, the UAE exhibited the most rapid increase in ASDR, escalating from 24.32 (95% UI: 4.07–60.28) to 80.08 (95% UI: 22.77–140.04), with an AAPC of 1.811 (95% UI: 1.694–1.928). This rapid rise in ASDR reflects a substantial increase in the rate of health loss due to ovarian cancer attributable to high BMI in the UAE.

We further evaluated the global and regional PAF of ovarian cancer attributable to high BMI, as presented in **Supplementary Figure S2** and **Supplementary Table S4**. Globally, the PAF for mortality was 9.32% (95%UI: 2.25%–16.62%), and for DALYs, it was 6.74% (95%UI: 1.31%–13.02%). Among all regions, the highest PAFs were observed in high-SDI and high-middle SDI areas, with particularly elevated values in North Africa and the Middle East, as well as Southern Sub-Saharan Africa. Targeted efforts to reduce BMI in these regions may be crucial for mitigating the burden of ovarian cancer attributable to high BMI.

### Correlation of ASMR and ASDR of ovarian cancer attributable to high BMI with SDI

**Figure 5:**
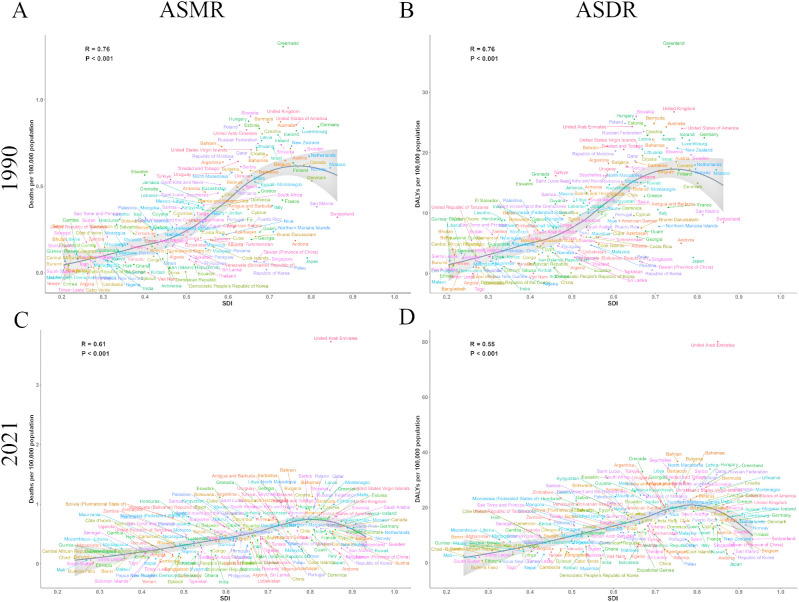
The correlation between SDI and burden of ovarian cancer attributable to high body-mass index in 204 countries and territories in 1990 and 2021. **(A)** The correlation between SDI and ASMR in 1990; **(B)** The correlation between SDI and ASDR in 1990; **(C)** The correlation between SDI and ASMR in 2021; **(D)** The correlation between SDI and ASDR in 2021. DALY, disability-adjusted life years; ASMR, age-standardized rates of mortality; ASDR, age-standardized rate of DALYs.

We conducted a systematic analysis to examine the complex relationship between SDI and the two key indicators of ovarian cancer burden attributable to high BMI, ASMR and ASDR. Notably, data from both 1990 and 2021 revealed the significant impact of SDI on the burden of ovarian cancer attributable to high BMI. Our findings showed a marked positive correlation between SDI and both ASMR and ASDR in 1990 (**Figure 5A, B**), with correlation coefficients of r = 0.76 (*P* < 0.001). Data from 2021 further substantiated this trend (**Figure 5C, D**), with ASMR (r = 0.61, *P* < 0.001) and ASDR (r = 0.55, *P* < 0.001) remaining positively correlated with SDI.

Both ASMR and ASDR increased progressively as SDI increased from 0.2 to 0.7. Peak values were attained within an SDI range of 0.7 to 0.8, followed by a slight decline at higher SDI levels. Based on these findings, we suggest that public health interventions focusing on ovarian cancer attributable to high BMI should be tailored according to the SDI levels of different countries and regions. Regions with an SDI range between 0.7 and 0.8 experienced the most significant disease burden, underscoring the need for policymakers to prioritize these areas when formulating targeted public health policies to address the impact of high BMI on ovarian cancer.

### Forecasting trends in ASMR and ASDR of ovarian cancer attributable to high BMI

**Figure 6:**
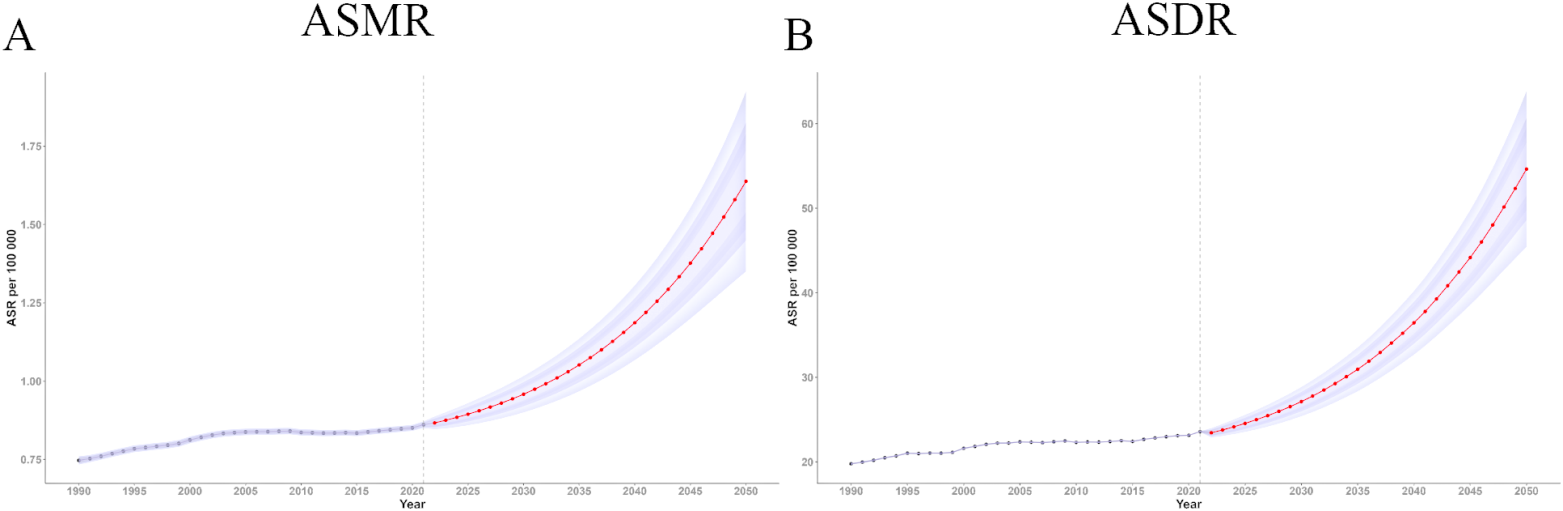
Trend of ASMR and ASDR of ovarian cancer attributable to high body-mass index from 2021 to 2050. **(A)** Trend of ASMR from 2021 to 2050; **(B)** Trend of ASDR from 2021 to 2050. DALY, disability-adjusted life years; ASMR, age-standardized rates of mortality; ASDR, age-standardized rate of DALYs.

Using the BAPC model in R, we projected the ASMR and ASDR for ovarian cancer attributable to high BMI from 2022 to 2050. The predictions indicate a sustained increase in disease burden over time. The estimated ASMR for 2022 was 0.87 (95% UI 0.85, 0.89), which increased significantly to 1.64 (95% UI 1.35, 1.93) by 2050, suggesting a substantial future increase in the mortality risk from ovarian cancer attributable to high BMI (**Figure 6A**).

Similarly, the ASDR for ovarian cancer attributable to high BMI showed a steady upward trend over time (**Figure 6B**). The BAPC model estimated an ASDR of 23.45 in 2022 (95% UI 22.89, 24.00), increasing to 54.63 (95% UI 45.41, 63.84) by 2050. These results highlight the escalating global burden of ovarian cancer attributable to high BMI and offer crucial insights to inform the development of public health policies aimed at mitigating the potentially growing disease burden in the future.

## Discussion

This study employed GBD 2021 data to explore the distribution and trends of the disease burden linked to ovarian cancer attributable to high BMI. Although the ASMR and ASDR for ovarian cancer attributable to high BMI exhibited minimal fluctuations from 1990 to 2021, the mortality and disease burden, measured as DALYs, showed a significant increase, which could be associated with demographic shifts, in particular, population aging. The results suggest that the public health impact of ovarian cancer is intensifying, especially in populations with elevated BMI levels.

Ovarian cancer remains a prevalent and highly lethal malignancy affecting women worldwide, with therapeutic resistance presenting a persistent clinical challenge (17–19). Statistical trends indicate a continuous increase in the age-standardized incidence of ovarian cancer from 1990 to 2021 (20). In China alone, data from 2019 estimated approximately 196,000 ovarian cancer patients, with 45,000 new diagnoses and 29,000 deaths. These statistics reflect a substantial and growing disease burden of ovarian cancer in China that is projected to increase at a rate surpassing the global average (12). Existing genetic testing methods are limited in their ability to effectively identify high-risk groups for early prevention (21). More importantly, large-scale clinical trials, such as UK Collaborative Trial of Ovarian Cancer Screening (UKCTOCS) have demonstrated that screening does not significantly reduce mortality rates, thereby discouraging widespread screening initiatives (22). Taken together, these factors contribute to the sustained and formidable disease burden of ovarian cancer, highlighting the urgent need for improved preventive and targeted intervention measures.

Recent studies have identified high BMI as a significant risk factor for ovarian cancer, with evidence demonstrating a positive correlation between increasing BMI and the likelihood of developing the disease (23, 24). Factors such as obesity and overweight have been shown to substantially increase both the incidence and mortality of ovarian cancer. Several investigations have proposed that effective weight management could alleviate the burden of gynecological cancers among Chinese women (25). However, contrasting findings indicate that obesity in ovarian cancer patients may have no significant impact on prognosis, with only a temporary improvement in three-year survival rates (26). This discrepancy highlights the complex relationship between high BMI and ovarian cancer outcomes, suggesting that while elevated BMI may be implicated in the pathogenesis of the disease, its influence on patient prognosis remains unclear and warrants further investigation (27). Therefore, addressing ovarian cancer attributable to high BMI should be a priority, including the development of effective public health interventions to mitigate the growing disease burden.

Our study revealed a significant positive correlation between the ASMR and ASDR of ovarian cancer attributable to high BMI and SDI, underscoring the critical role of socioeconomic factors in the prevalence of this disease. The burden of ovarian cancer attributable to high BMI was particularly pronounced in regions with SDI values between 0.7 and 0.8. This trend mirrored the global pattern of rising disease burden attributable to high BMI followed by a decline, especially evident in regions with SDI ranging from 0.75 to 0.85, where the burden was most substantial (28). This occurrence may be linked to disparities in economic development and the inconsistent implementation of health policies across these areas. In regions with high SDI, socioeconomic development may initially contribute to a greater ovarian cancer burden attributable to high BMI. However, once SDI surpasses a threshold (~0.7–0.8), the primary determinants shift towards factors characteristic of high-SDI settings, such as improved healthcare access, nutritional standards, health literacy, and education, leading to a net reduction in disease burden. Typically, regions with higher SDI tend to benefit from more advanced healthcare systems. For instance, statistical data from China between 2001 and 2020 demonstrate that economically developed cities have a relatively higher number of physicians and greater availability of hospital beds, contributing to a richer healthcare resource pool (29). However, the increase in SDI is often accompanied by lifestyle changes that potentially lead to a higher prevalence of obesity, thereby elevating the risk of ovarian cancer. Therefore, despite the advantages conferred by improved healthcare resources, the burden of ovarian cancer attributable to high BMI remains a pressing issue in higher SDI settings.

An ASMR of 1.64 per 100,000 for ovarian cancer attributable to high BMI was predicted by 2050. The projection aligns with escalating global obesity, with the number of overweight/obese adults projected to reach 3.8 billion by 2050 (11). Concurrently, the overall burden of ovarian cancer is anticipated to increase, with a predicted ASMR of 2.54 per 100,000 by 2030 (12) and continued increase in total case numbers by 2050 (13). Collectively, these trends underscore the expanding influence of high BMI on disease burden and highlight the urgent necessity for targeted interventions. In response to the growing concern, on March 10, 2025, China’s National Health Commission launched a three-year initiative titled “Weight Management Year”. This program prioritizes the promotion of healthy lifestyles and enhanced prevention and control of chronic diseases. Future policies are advocated to foster environments that support healthy behaviors, including improving access to nutritious food, expanding the coverage of safe and convenient sports infrastructure, and limiting the marketing of unhealthy food products. In addition, improved health education, gynecological consultations and routine health examinations are advocated, with particular emphasis on raising awareness of the risks of ovarian cancer and the importance of weight management for overweight and obese women.

While this study provides a comprehensive global assessment of the ovarian cancer burden attributable to elevated BMI by drawing on 2021 GBD data with projections to 2050, several limitations warrant consideration. First, while BMI serves as a well-established metric for evaluation of population-level risk, its application in this work does not fully characterize individual variations in body composition or adiposity distribution. Future research should integrate more precise indicators of adiposity, such as waist-hip ratio and visceral adiposity assessments. Second, the accuracy of the data may be limited by reporting bias, which could introduce potential systematic errors. In regions with underdeveloped cancer registry systems, systematic underreporting or omission of ovarian cancer cases leads to underestimation of the true disease burden, particularly in low-resource settings. This could disproportionately affect data obtained from SDI-stratified regions, potentially obscuring genuine disparities in healthcare access and data quality. Third, the absence of longitudinal cohort data specific to high BMI populations introduced a critical methodological constraint. Without long-term follow-up tracking of BMI trajectories and ovarian cancer outcomes, our analysis could not account for latency effects or cumulative exposure risks. This limitation may induce random errors when quantifying attributable fractions, thereby reducing the precision of projecting future burdens. Fourth, the GBD 2021 modelling framework did not adequately account for the profound disruptions caused by the COVID-19 pandemic (30). Delays in cancer screening, diagnosis, and treatment during this period (31, 32) may have introduced temporal confounding factors into ASMR/ASDR estimates, particularly compromising the accuracy of short-term trends and 2050 projections, with pandemic-related mortality peaks potentially misinterpreted as indicative of long-term trends.

## Conclusion

In summary, the current study highlights the ongoing upward trend in the global burden of ovarian cancer attributable to high BMI. As global obesity rates continue to rise, the associated risk of ovarian cancer is expected to increase correspondingly. Public health policies should therefore focus on high-BMI populations, particularly in regions with high SDI and those within an SDI range of 0.7 to 0.8, where the disease burden is most pronounced. Targeted prevention and intervention measures should include reducing obesogenic environmental factors and improving ovarian cancer risk assessment strategies for women with elevated BMI.

## Data Availability

The original contributions presented in the study are included in the article/**Supplementary Material**. Further inquiries can be directed to the corresponding author.
