# Supplementary material for: Global, regional, and national burden of ovarian cancer attributable to high body mass index, 1990–2021: insights from the global burden of disease study 2021
Source: Front Oncol. 2025 Oct 31;15:1568716. doi: 10.3389/fonc.2025.1568716 (PMC12615190; doi:10.3389/fonc.2025.1568716)
Supplement: Supplementary file 1 [file DataSheet1.docx]

Supplementary_Materials

The model adopts a Bayesian hierarchical framework and estimates parameters via the Integrated Nested Laplace Approximation (INLA) method.

Figure S1 Joinpoint regression analysis of ASMR and ASDR of ovarian cancer attributable to high body-mass index from 1990 to 2021.

Figure S2 The PAF of death and DALYs of ovarian cancer attributable to high body-mass index in region pattern in2021. (A) Death; (B) DALYs. PAF, population attributable fraction; DALY, disability-adjusted life years.

Table S1 The global number and age-standardized rate of deaths and DALYs in ovarian cancer attributable to high body-mass index from 1990 to 2021

Table S2 The ASMR, ASDR and their AAPC of ovarian cancer attributable to high body-mass index in 5 SDI and 21 GBD regions in 1990 and 2021.

Table S3 The ASMR, ASDR and their AAPC of ovarian cancer attributable to high body-mass index in 204 countries or territories in 1990 and 2021

Table S4 The Population Attributable Fraction of ovarian cancer attributable to high body-mass index in region pattern in 2021.

We used the BAPC package in R to model and forecast age-specific prevalence of high BMI-attributable ovarian cancer from 1990 to 2021, with projections extending to 2050. The model adopts a Bayesian hierarchical framework and estimates parameters via the Integrated Nested Laplace Approximation (INLA) method. The model specifications are as follows:(1) The age effect was modeled using a second-order random walk (RW2) to capture smooth transitions between adjacent age groups. (2) The period effect was specified as a first-order random walk (RW1) to reflect year-to-year variations in prevalence. (3) The cohort effect was modeled using an RW2 process to capture intergenerational trends. (4) Overdispersion was accounted for using an independent and identically distributed (IID) structure to address extra variability in the data. (5) All random effects were assigned log-gamma priors (loggamma with parameters c(1, 0.00005) or c(1, 0.005)), following the weakly informative priors recommended for BAPC modeling. (6) The standard population weights were based on the WHO World Standard Population (2000–2025) to calculate age-standardized rates (ASR). (7) The projection horizon was set to 29 years (2022–2050), and we also enabled retrospective prediction (retro = TRUE) to assess the model's fitting stability. (8) We presented multi-level credible intervals (50% to 95%) in the plots to convey prediction uncertainty. All assumptions and model specifications were guided by the work of Riebler and Held on the BAPC model [1].

[1] Riebler, A. and L. Held, Projecting the future burden of cancer: Bayesian age-period-cohort analysis with integrated nested Laplace approximations. Biom J, 2017. 59(3): p. 531-549.


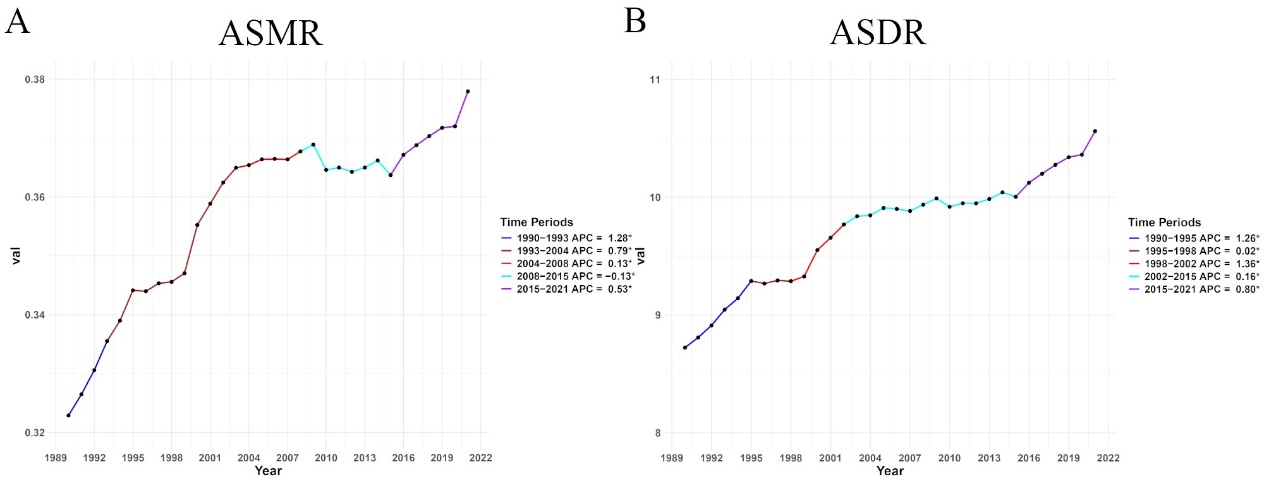


Figure S1 Joinpoint regression analysis of ASMR and ASDR of ovarian cancer attributable to high body-mass index from 1990 to 2021. (A) ASMR; (B) ASDR. DALY, disability-adjusted life years; ASMR, age-standardized rates of mortality; ASDR, age-standardized rate of DALYs.


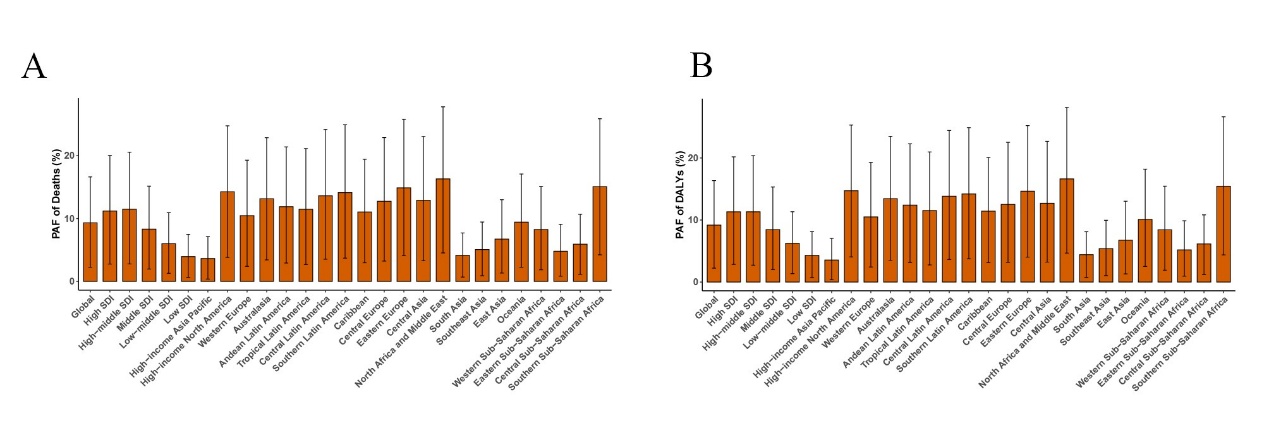


Figure S2 The PAF of death and DALYs of ovarian cancer attributable to high body-mass index in region pattern in2021. (A) Death; (B) DALYs. PAF, population attributable fraction; DALY, disability-adjusted life years.

Table S1 The global number and age-standardized rate of deaths and DALYs in ovarian cancer attributable to high body-mass index from 1990 to 2021

| Year | Death | | DALY | |
| --- | --- | --- | --- | --- |
|  | Number | ASR | Number | ASR |
| 1990 | 6850 (1423, 12865) | 0.32 (0.07, 0.61) | 188874 (38401, 355691) | 8.72 (1.78, 16.41) |
| 1991 | 7075 (1475, 13236) | 0.33 (0.07, 0.61) | 194709 (39804, 364556) | 8.81 (1.81, 16.47) |
| 1992 | 7317 (1522, 13617) | 0.33 (0.07, 0.62) | 201054 (40909, 374020) | 8.91 (1.82, 16.56) |
| 1993 | 7583 (1582, 14164) | 0.34 (0.07, 0.63) | 208285 (42755, 389221) | 9.05 (1.86, 16.89) |
| 1994 | 7825 (1633, 14561) | 0.34 (0.07, 0.63) | 214874 (44226, 400213) | 9.14 (1.89, 17.01) |
| 1995 | 8111 (1701, 15055) | 0.34 (0.07, 0.64) | 222715 (46222, 414794) | 9.29 (1.93, 17.28) |
| 1996 | 8283 (1738, 15384) | 0.34 (0.07, 0.64) | 226979 (47291, 422075) | 9.27 (1.93, 17.22) |
| 1997 | 8504 (1786, 15711) | 0.35 (0.07, 0.64) | 232677 (48317, 431617) | 9.29 (1.93, 17.22) |
| 1998 | 8703 (1832, 16067) | 0.35 (0.07, 0.64) | 237650 (49574, 438466) | 9.29 (1.94, 17.11) |
| 1999 | 8946 (1883, 16510) | 0.35 (0.07, 0.64) | 244186 (50819, 451005) | 9.33 (1.95, 17.20) |
| 2000 | 9369 (1996, 17283) | 0.36 (0.08, 0.66) | 255687 (53697, 470165) | 9.55 (2.01, 17.55) |
| 2001 | 9694 (2081, 17815) | 0.36 (0.08, 0.66) | 264688 (55975, 485780) | 9.65 (2.05, 17.69) |
| 2002 | 10032 (2175, 18352) | 0.36 (0.08, 0.66) | 274271 (58782, 501431) | 9.77 (2.10, 17.84) |
| 2003 | 10347 (2272, 18949) | 0.36 (0.08, 0.67) | 282926 (61249, 518582) | 9.84 (2.14, 17.99) |
| 2004 | 10623 (2338, 19433) | 0.37 (0.08, 0.67) | 290265 (63274, 531695) | 9.85 (2.15, 18.00) |
| 2005 | 10924 (2408, 20031) | 0.37 (0.08, 0.67) | 299472 (65700, 552640) | 9.91 (2.18, 18.25) |
| 2006 | 11216 (2472, 20514) | 0.37 (0.08, 0.67) | 306967 (67742, 563395) | 9.90 (2.20, 18.14) |
| 2007 | 11519 (2560, 21023) | 0.37 (0.08, 0.67) | 314468 (69929, 573924) | 9.88 (2.21, 18.02) |
| 2008 | 11880 (2662, 21664) | 0.37 (0.08, 0.67) | 324693 (72900, 593790) | 9.94 (2.24, 18.16) |
| 2009 | 12252 (2753, 22417) | 0.37 (0.08, 0.67) | 335249 (75707, 611802) | 9.99 (2.26, 18.22) |
| 2010 | 12448 (2804, 22628) | 0.36 (0.08, 0.66) | 341812 (77583, 618552) | 9.92 (2.25, 17.94) |
| 2011 | 12812 (2912, 23089) | 0.36 (0.08, 0.66) | 352038 (80165, 637997) | 9.95 (2.27, 18.02) |
| 2012 | 13152 (3004, 23778) | 0.36 (0.08, 0.66) | 361430 (82450, 651844) | 9.95 (2.27, 17.93) |
| 2013 | 13551 (3121, 24243) | 0.36 (0.08, 0.65) | 372430 (86018, 664516) | 9.98 (2.31, 17.81) |
| 2014 | 13984 (3244, 25181) | 0.37 (0.08, 0.66) | 384454 (89344, 689098) | 10.04 (2.33, 17.99) |
| 2015 | 14273 (3324, 25557) | 0.36 (0.08, 0.65) | 392898 (90769, 699726) | 10.00 (2.31, 17.81) |
| 2016 | 14809 (3479, 26467) | 0.37 (0.09, 0.66) | 407694 (95442, 721706) | 10.12 (2.37, 17.92) |
| 2017 | 15286 (3601, 27411) | 0.37 (0.09, 0.66) | 420984 (98832, 752622) | 10.20 (2.39, 18.24) |
| 2018 | 15770 (3741, 28235) | 0.37 (0.09, 0.66) | 434455 (102526, 778562) | 10.27 (2.42, 18.41) |
| 2019 | 16258 (3848, 29127) | 0.37 (0.09, 0.67) | 447564 (105853, 799133) | 10.34 (2.44, 18.47) |
| 2020 | 16682 (3980, 29978) | 0.37 (0.09, 0.67) | 458691 (109300, 816260) | 10.36 (2.46, 18.44) |
| 2021 | 17344 (4141, 30810) | 0.38 (0.09, 0.67) | 477248 (113449, 840002) | 10.56 (2.50, 18.57) |

Table S2 The ASMR, ASDR and their AAPC of ovarian cancer attributable to high body-mass index in 5 SDI and 21 GBD regions in 1990 and 2021.

| Location | ASMR | | | ASDR | | |
| --- | --- | --- | --- | --- | --- | --- |
|  | 1990 | 2021 | AAPC | 1990 | 2021 | AAPC |
| Global | 0.32 (0.07, 0.61) | 0.38 (0.09, 0.67) | 0.002(0.002,0.002) | 8.72 (1.78, 16.41) | 10.56 (2.50, 18.57) | 0.058(0.054,0.062) |
| High SDI | 0.61 (0.13, 1.15) | 0.57 (0.14, 1.01) | -0.002(-0.002,-0.001) | 16.78 (3.57, 31.36) | 15.13 (3.79, 26.82) | -0.057(-0.063,-0.051) |
| High-middle SDI | 0.40 (0.09, 0.75) | 0.48 (0.12, 0.85) | 0.002(0.002,0.003) | 11.71 (2.49, 21.84) | 13.54 (3.26, 24.13) | 0.058(0.046,0.070) |
| Middle SDI | 0.10 (0.01, 0.19) | 0.26 (0.06, 0.48) | 0.005(0.005,0.005) | 2.99 (0.36, 5.90) | 8.25 (1.95, 15.09) | 0.170(0.169,0.171) |
| Low-middle SDI | 0.06 (0.00, 0.12) | 0.22 (0.04, 0.41) | 0.005(0.005,0.005) | 1.78 (0.13, 3.74) | 6.96 (1.43, 12.58) | 0.167(0.166,0.169) |
| Low SDI | 0.05 (-0.00, 0.11) | 0.15 (0.02, 0.29) | 0.003(0.003,0.003) | 1.60 (0.01, 3.56) | 4.68 (0.74, 9.04) | 0.100(0.099,0.101) |
| Andean Latin America | 0.16 (0.03, 0.33) | 0.50 (0.12, 0.97) | 0.011(0.011,0.011) | 5.20 (0.93, 10.80) | 15.55 (3.86, 29.85) | 0.332(0.322,0.341) |
| Australasia | 0.85 (0.18, 1.58) | 0.62 (0.16, 1.10) | -0.009(-0.010,-0.007) | 23.68 (5.00, 43.97) | 15.67 (4.12, 27.86) | -0.263(-0.309,-0.217) |
| Caribbean | 0.22 (0.05, 0.42) | 0.46 (0.11, 0.84) | 0.008(0.008,0.008) | 6.81 (1.44, 12.82) | 14.23 (3.43, 26.10) | 0.243(0.234,0.252) |
| Central Asia | 0.29 (0.06, 0.54) | 0.53 (0.13, 0.95) | 0.008(0.007,0.008) | 9.08 (2.00, 16.79) | 15.77 (3.76, 28.49) | 0.223(0.213,0.233) |
| Central Europe | 0.70 (0.16, 1.30) | 0.94 (0.24, 1.71) | 0.007(0.007,0.008) | 20.59 (4.65, 38.13) | 25.65 (6.53, 46.23) | 0.155(0.141,0.169) |
| Central Latin America | 0.32 (0.07, 0.59) | 0.67 (0.19, 1.21) | 0.011(0.011,0.012) | 9.51 (2.09, 17.81) | 20.87 (5.99, 37.44) | 0.373(0.363,0.384) |
| Central Sub-Saharan Africa | 0.05 (0.00, 0.10) | 0.18 (0.03, 0.37) | 0.004(0.004,0.005) | 1.35 (0.01, 3.08) | 5.55 (0.92, 11.34) | 0.136(0.134,0.137) |
| East Asia | 0.03 (-0.02, 0.08) | 0.16 (0.03, 0.33) | 0.004(0.004,0.004) | 0.97 (-0.47, 2.63) | 4.86 (0.96, 9.99) | 0.127(0.126,0.128) |
| Eastern Europe | 0.68 (0.16, 1.21) | 0.89 (0.24, 1.54) | 0.006(0.005,0.007) | 21.36 (4.99, 37.80) | 26.48 (6.87, 45.59) | 0.162(0.109,0.216) |
| Eastern Sub-Saharan Africa | 0.08 (-0.00, 0.17) | 0.25 (0.04, 0.49) | 0.006(0.006,0.006) | 2.48 (0.02, 5.26) | 7.84 (1.32, 15.40) | 0.173(0.172,0.174) |
| High-income Asia Pacific | 0.07 (-0.01, 0.17) | 0.12 (0.01, 0.24) | 0.001(0.001,0.001) | 2.29 (-0.28, 5.19) | 3.50 (0.38, 6.99) | 0.039(0.038,0.041) |
| High-income North America | 0.83 (0.20, 1.52) | 0.78 (0.21, 1.34) | -0.002(-0.003,-0.002) | 23.36 (5.74, 42.27) | 20.45 (5.66, 35.38) | -0.109(-0.121,-0.096) |
| North Africa and Middle East | 0.24 (0.05, 0.49) | 0.51 (0.15, 0.89) | 0.009(0.008,0.009) | 7.27 (1.49, 14.33) | 14.62 (4.22, 25.60) | 0.236(0.234,0.237) |
| Oceania | 0.11 (0.02, 0.22) | 0.19 (0.05, 0.34) | 0.003(0.002,0.003) | 3.41 (0.66, 6.95) | 5.93 (1.54, 11.06) | 0.081(0.078,0.084) |
| South Asia | 0.03 (-0.01, 0.07) | 0.16 (0.03, 0.30) | 0.004(0.004,0.004) | 0.98 (-0.16, 2.23) | 5.11 (0.82, 9.43) | 0.135(0.134,0.136) |
| Southeast Asia | 0.05 (-0.00, 0.10) | 0.20 (0.04, 0.38) | 0.005(0.005,0.005) | 1.67 (-0.06, 3.64) | 6.82 (1.27, 12.97) | 0.167(0.166,0.168) |
| Southern Latin America | 0.59 (0.14, 1.12) | 0.68 (0.18, 1.20) | 0.003(0.002,0.004) | 16.81 (3.87, 31.61) | 19.48 (5.21, 34.22) | 0.086(0.064,0.108) |
| Southern Sub-Saharan Africa | 0.36 (0.09, 0.68) | 0.81 (0.22, 1.43) | 0.015(0.014,0.015) | 10.58 (2.68, 19.98) | 23.20 (6.41, 41.25) | 0.401(0.391,0.411) |
| Tropical Latin America | 0.28 (0.06, 0.55) | 0.46 (0.11, 0.84) | 0.005(0.005,0.006) | 8.52 (1.78, 16.56) | 13.52 (3.18, 24.66) | 0.157(0.150,0.165) |
| Western Europe | 0.63 (0.13, 1.20) | 0.58 (0.14, 1.06) | -0.002(-0.002,-0.001) | 17.18 (3.53, 32.53) | 14.70 (3.42, 26.90) | -0.075(-0.085,-0.066) |
| Western Sub-Saharan Africa | 0.07 (0.01, 0.14) | 0.21 (0.05, 0.41) | 0.004(0.004,0.005) | 2.18 (0.34, 4.04) | 6.12 (1.30, 11.80) | 0.125(0.124,0.126) |

Table S3 The ASMR, ASDR and their AAPC of ovarian cancer attributable to high body-mass index in 204 countries or territories in 1990 and 2021

| Location | ASMR | | | ASDR | | |
| --- | --- | --- | --- | --- | --- | --- |
|  | 1990 | 2021 | AAPC | 1990 | 2021 | AAPC |
| American Samoa | 0.26 (0.07, 0.49) | 0.56 (0.16, 1.04) | 0.010(0.010,0.010) | 8.73 (2.24, 16.36) | 18.19 (5.36, 33.95) | 0.318(0.304,0.332) |
| Antigua and Barbuda | 0.37 (0.07, 0.68) | 0.84 (0.20, 1.51) | 0.014(0.013,0.016) | 11.40 (2.34, 21.50) | 24.19 (5.92, 44.27) | 0.395(0.355,0.436) |
| Arab Republic of Egypt | 0.15 (0.04, 0.44) | 0.59 (0.18, 1.04) | 0.014(0.014,0.015) | 4.46 (1.08, 12.19) | 16.45 (4.98, 28.88) | 0.386(0.377,0.394) |
| Argentine Republic | 0.64 (0.15, 1.21) | 0.71 (0.19, 1.27) | 0.002(0.002,0.003) | 18.20 (4.13, 34.54) | 20.15 (5.44, 35.71) | 0.067(0.050,0.083) |
| Australia | 0.87 (0.19, 1.63) | 0.63 (0.17, 1.13) | -0.010(-0.013,-0.007) | 24.26 (5.12, 45.55) | 16.08 (4.28, 28.70) | -0.271(-0.321,-0.221) |
| Barbados | 0.59 (0.13, 1.16) | 0.93 (0.26, 1.71) | 0.011(0.010,0.012) | 18.40 (4.02, 35.32) | 27.53 (8.23, 49.29) | 0.294(0.264,0.325) |
| Belize | 0.21 (0.05, 0.38) | 0.40 (0.12, 0.70) | 0.006(0.006,0.007) | 6.72 (1.63, 11.78) | 12.70 (3.77, 21.65) | 0.203(0.188,0.219) |
| Bermuda | 0.87 (0.18, 1.70) | 0.98 (0.28, 1.78) | 0.004(0.003,0.006) | 24.80 (5.17, 47.57) | 27.35 (8.23, 49.10) | 0.094(0.058,0.129) |
| Bolivarian Republic of Venezuela | 0.13 (0.03, 0.24) | 0.62 (0.17, 1.14) | 0.016(0.015,0.016) | 3.82 (0.86, 7.11) | 19.10 (5.41, 35.38) | 0.494(0.466,0.523) |
| Bosnia and Herzegovina | 0.41 (0.09, 0.78) | 0.72 (0.18, 1.32) | 0.010(0.009,0.010) | 12.24 (2.66, 23.00) | 20.30 (5.17, 37.34) | 0.252(0.239,0.266) |
| Brunei Darussalam | 0.21 (0.01, 0.47) | 0.56 (0.13, 1.00) | 0.011(0.011,0.012) | 7.54 (0.62, 16.50) | 19.17 (4.56, 34.83) | 0.382(0.374,0.391) |
| Burkina Faso | 0.01 (-0.01, 0.03) | 0.02 (-0.01, 0.06) | 0.000(0.000,0.000) | 0.28 (-0.26, 0.96) | 0.82 (-0.15, 2.21) | 0.018(0.017,0.018) |
| Canada | 0.62 (0.14, 1.16) | 0.62 (0.16, 1.12) | -0.000(-0.001,0.001) | 17.21 (3.92, 32.32) | 16.04 (4.19, 28.76) | -0.033(-0.053,-0.012) |
| Central African Republic | 0.05 (-0.00, 0.11) | 0.14 (0.02, 0.30) | 0.003(0.003,0.003) | 1.48 (0.03, 3.68) | 4.43 (0.78, 9.57) | 0.096(0.094,0.097) |
| Commonwealth of Dominica | 0.37 (0.08, 0.71) | 0.54 (0.15, 0.99) | 0.005(0.004,0.006) | 10.17 (2.22, 19.47) | 15.61 (4.28, 29.11) | 0.174(0.159,0.190) |
| Commonwealth of the Bahamas | 0.68 (0.16, 1.33) | 1.13 (0.30, 2.07) | 0.016(0.015,0.017) | 22.17 (5.14, 43.30) | 35.80 (9.60, 65.14) | 0.478(0.448,0.508) |
| Cook Islands | 0.15 (0.03, 0.29) | 0.25 (0.07, 0.44) | 0.003(0.003,0.003) | 4.88 (1.19, 9.17) | 7.91 (2.30, 14.00) | 0.097(0.093,0.102) |
| Czech Republic | 0.80 (0.18, 1.51) | 0.86 (0.21, 1.63) | 0.003(0.002,0.005) | 22.91 (5.14, 43.03) | 22.69 (5.47, 42.86) | -0.008(-0.055,0.040) |
| Democratic People's Republic of Korea | 0.01 (-0.02, 0.03) | 0.07 (0.00, 0.18) | 0.002(0.002,0.002) | 0.10 (-0.64, 0.99) | 1.60 (-0.23, 4.49) | 0.049(0.048,0.049) |
| Democratic Republic of Sao Tome and Principe | 0.21 (0.04, 0.40) | 0.49 (0.12, 1.04) | 0.009(0.009,0.009) | 6.44 (1.23, 12.69) | 14.88 (3.61, 31.55) | 0.274(0.269,0.279) |
| Democratic Republic of Timor-Leste | -0.00 (-0.02, 0.01) | 0.03 (-0.01, 0.10) | 0.001(0.001,0.001) | -0.04 (-0.49, 0.54) | 1.39 (-0.32, 3.75) | 0.046(0.046,0.046) |
| Democratic Republic of the Congo | 0.04 (-0.00, 0.09) | 0.17 (0.03, 0.36) | 0.004(0.004,0.004) | 1.05 (-0.09, 2.63) | 4.93 (0.81, 10.45) | 0.124(0.123,0.125) |
| Democratic Socialist Republic of Sri Lanka | 0.07 (0.01, 0.14) | 0.19 (0.04, 0.39) | 0.004(0.004,0.004) | 2.14 (0.18, 4.63) | 5.88 (1.25, 12.32) | 0.118(0.114,0.122) |
| Dominican Republic | 0.08 (0.01, 0.18) | 0.23 (0.04, 0.44) | 0.005(0.005,0.005) | 2.88 (0.44, 6.11) | 7.46 (1.58, 14.29) | 0.148(0.146,0.150) |
| Eastern Republic of Uruguay | 0.58 (0.13, 1.10) | 0.74 (0.19, 1.34) | 0.005(0.004,0.006) | 16.20 (3.66, 30.99) | 20.97 (5.20, 37.60) | 0.160(0.137,0.183) |
| Federal Democratic Republic of Ethiopia | 0.05 (-0.01, 0.14) | 0.10 (-0.00, 0.21) | 0.002(0.002,0.002) | 1.61 (-0.31, 4.96) | 3.06 (0.05, 6.42) | 0.047(0.046,0.047) |
| Federal Democratic Republic of Nepal | 0.02 (-0.01, 0.06) | 0.08 (0.00, 0.20) | 0.002(0.002,0.002) | 0.64 (-0.29, 1.96) | 3.02 (0.19, 7.15) | 0.078(0.077,0.078) |
| Federal Republic of Germany | 0.84 (0.17, 1.58) | 0.61 (0.14, 1.14) | -0.007(-0.008,-0.007) | 22.47 (4.53, 42.25) | 15.57 (3.61, 28.99) | -0.217(-0.232,-0.201) |
| Federal Republic of Nigeria | 0.07 (0.01, 0.15) | 0.26 (0.05, 0.50) | 0.006(0.006,0.006) | 2.03 (0.23, 4.17) | 6.96 (1.42, 13.67) | 0.157(0.155,0.158) |
| Federal Republic of Somalia | 0.07 (-0.00, 0.18) | 0.16 (0.01, 0.35) | 0.003(0.003,0.003) | 2.63 (0.10, 6.51) | 5.70 (0.62, 12.23) | 0.099(0.099,0.100) |
| Federated States of Micronesia | 0.22 (0.05, 0.44) | 0.50 (0.11, 1.05) | 0.009(0.009,0.009) | 7.57 (1.78, 15.23) | 16.53 (3.87, 34.73) | 0.289(0.284,0.294) |
| Federative Republic of Brazil | 0.29 (0.06, 0.55) | 0.46 (0.11, 0.85) | 0.005(0.005,0.006) | 8.61 (1.80, 16.73) | 13.58 (3.20, 24.81) | 0.157(0.149,0.166) |
| French Republic | 0.42 (0.07, 0.77) | 0.50 (0.12, 0.91) | 0.003(0.003,0.003) | 10.93 (1.82, 19.79) | 12.30 (2.83, 22.26) | 0.051(0.046,0.056) |
| Gabonese Republic | 0.25 (0.05, 0.48) | 0.57 (0.14, 1.13) | 0.010(0.010,0.011) | 7.59 (1.42, 14.58) | 17.10 (4.40, 33.65) | 0.305(0.300,0.309) |
| Georgia | 0.19 (0.05, 0.38) | 1.03 (0.24, 1.97) | 0.028(0.027,0.030) | 6.09 (1.47, 11.96) | 30.01 (6.89, 57.51) | 0.804(0.766,0.841) |
| Grand Duchy of Luxembourg | 0.84 (0.17, 1.57) | 0.69 (0.17, 1.28) | -0.005(-0.007,-0.004) | 22.07 (4.49, 41.58) | 16.80 (4.23, 31.29) | -0.183(-0.217,-0.149) |
| Greenland | 1.31 (0.32, 2.47) | 0.92 (0.23, 1.70) | -0.012(-0.014,-0.011) | 37.54 (9.06, 70.32) | 28.79 (7.15, 53.83) | -0.269(-0.309,-0.229) |
| Grenada | 0.47 (0.09, 1.01) | 1.05 (0.26, 1.97) | 0.019(0.018,0.020) | 15.90 (2.94, 34.29) | 32.98 (8.57, 60.81) | 0.570(0.545,0.594) |
| Guam | 0.24 (0.05, 0.49) | 0.40 (0.10, 0.74) | 0.005(0.003,0.006) | 7.01 (1.50, 14.73) | 14.18 (3.66, 25.90) | 0.226(0.199,0.253) |
| Hashemite Kingdom of Jordan | 0.36 (0.09, 0.71) | 0.67 (0.20, 1.22) | 0.009(0.009,0.010) | 10.66 (2.65, 20.65) | 18.53 (5.48, 33.87) | 0.246(0.233,0.259) |
| Hellenic Republic | 0.46 (0.09, 0.90) | 0.70 (0.17, 1.29) | 0.008(0.007,0.009) | 12.78 (2.52, 24.79) | 18.63 (4.53, 34.01) | 0.198(0.180,0.216) |
| Hungary | 0.88 (0.21, 1.55) | 0.92 (0.25, 1.66) | 0.001(-0.000,0.002) | 25.54 (5.92, 45.27) | 25.94 (7.06, 46.62) | -0.011(-0.045,0.022) |
| Independent State of Papua New Guinea | 0.05 (0.00, 0.12) | 0.10 (0.02, 0.23) | 0.002(0.002,0.002) | 1.69 (0.18, 4.16) | 3.65 (0.65, 8.05) | 0.064(0.062,0.066) |
| Independent State of Samoa | 0.37 (0.10, 0.69) | 0.64 (0.16, 1.20) | 0.009(0.008,0.009) | 12.06 (3.55, 22.19) | 20.35 (5.41, 39.33) | 0.274(0.266,0.282) |
| Ireland | 0.80 (0.17, 1.53) | 0.72 (0.17, 1.32) | -0.003(-0.005,-0.002) | 22.84 (4.79, 43.51) | 18.49 (4.33, 33.70) | -0.149(-0.187,-0.111) |
| Islamic Republic of Afghanistan | 0.13 (0.02, 0.41) | 0.23 (0.04, 0.61) | 0.003(0.003,0.003) | 4.28 (0.55, 13.79) | 7.49 (1.45, 19.39) | 0.105(0.104,0.106) |
| Islamic Republic of Iran | 0.11 (0.02, 0.23) | 0.35 (0.10, 0.62) | 0.007(0.007,0.008) | 3.47 (0.67, 6.99) | 10.21 (3.08, 18.15) | 0.213(0.207,0.219) |
| Islamic Republic of Mauritania | 0.13 (0.03, 0.24) | 0.27 (0.06, 0.52) | 0.004(0.004,0.005) | 3.77 (0.78, 7.29) | 7.85 (1.81, 15.57) | 0.130(0.126,0.134) |
| Islamic Republic of Pakistan | 0.08 (-0.01, 0.20) | 0.45 (0.09, 0.94) | 0.012(0.012,0.012) | 2.80 (-0.17, 6.49) | 13.97 (2.75, 28.75) | 0.365(0.362,0.367) |
| Jamaica | 0.42 (0.09, 0.77) | 0.79 (0.22, 1.43) | 0.013(0.012,0.013) | 13.34 (2.89, 23.96) | 24.48 (6.95, 44.00) | 0.390(0.367,0.414) |
| Japan | 0.09 (-0.01, 0.19) | 0.11 (0.01, 0.23) | 0.001(0.001,0.001) | 2.65 (-0.24, 5.97) | 3.38 (0.27, 6.98) | 0.024(0.022,0.025) |
| Kingdom of Bahrain | 0.73 (0.16, 1.45) | 1.35 (0.41, 2.54) | 0.020(0.020,0.021) | 21.03 (4.82, 41.91) | 36.75 (11.28, 67.96) | 0.507(0.492,0.521) |
| Kingdom of Belgium | 0.65 (0.13, 1.22) | 0.54 (0.12, 0.99) | -0.004(-0.004,-0.003) | 17.42 (3.44, 32.57) | 13.54 (2.96, 24.34) | -0.146(-0.170,-0.122) |
| Kingdom of Bhutan | 0.12 (0.01, 0.28) | 0.27 (0.05, 0.63) | 0.005(0.005,0.005) | 4.01 (0.48, 9.29) | 8.74 (1.60, 20.81) | 0.155(0.153,0.157) |
| Kingdom of Cambodia | 0.03 (-0.01, 0.08) | 0.13 (0.00, 0.30) | 0.003(0.003,0.003) | 0.89 (-0.32, 2.66) | 4.39 (0.12, 10.25) | 0.114(0.114,0.115) |
| Kingdom of Denmark | 0.53 (0.10, 1.00) | 0.59 (0.13, 1.08) | 0.002(0.001,0.003) | 14.96 (2.77, 28.67) | 14.59 (3.12, 25.87) | -0.011(-0.043,0.021) |
| Kingdom of Eswatini | 0.56 (0.13, 1.22) | 1.11 (0.25, 2.25) | 0.018(0.017,0.018) | 15.43 (3.44, 32.96) | 30.72 (6.82, 62.88) | 0.491(0.478,0.505) |
| Kingdom of Lesotho | 0.31 (0.07, 0.67) | 0.79 (0.18, 1.68) | 0.016(0.015,0.016) | 8.82 (1.93, 19.09) | 22.49 (5.13, 47.89) | 0.441(0.429,0.454) |
| Kingdom of Morocco | 0.17 (0.03, 0.34) | 0.40 (0.10, 0.77) | 0.007(0.007,0.007) | 5.03 (0.93, 10.30) | 12.01 (2.99, 23.03) | 0.225(0.224,0.226) |
| Kingdom of Norway | 0.62 (0.11, 1.17) | 0.55 (0.12, 1.04) | -0.002(-0.003,-0.001) | 17.30 (3.31, 32.75) | 13.71 (3.03, 25.57) | -0.113(-0.135,-0.091) |
| Kingdom of Saudi Arabia | 0.24 (0.05, 0.51) | 0.61 (0.19, 1.21) | 0.012(0.012,0.012) | 7.17 (1.55, 15.46) | 17.92 (5.37, 36.38) | 0.349(0.345,0.354) |
| Kingdom of Spain | 0.48 (0.10, 0.89) | 0.56 (0.14, 1.00) | 0.003(0.002,0.003) | 13.65 (2.75, 25.14) | 14.77 (3.80, 26.79) | 0.038(0.023,0.053) |
| Kingdom of Sweden | 0.71 (0.14, 1.36) | 0.53 (0.12, 1.00) | -0.005(-0.006,-0.004) | 19.65 (4.02, 37.81) | 13.03 (2.99, 24.57) | -0.194(-0.215,-0.173) |
| Kingdom of Thailand | 0.08 (0.00, 0.17) | 0.34 (0.08, 0.67) | 0.009(0.008,0.009) | 2.67 (0.11, 5.60) | 11.52 (2.70, 22.74) | 0.288(0.285,0.291) |
| Kingdom of Tonga | 0.22 (0.05, 0.39) | 0.42 (0.12, 0.78) | 0.006(0.006,0.006) | 7.18 (1.78, 12.58) | 13.16 (3.94, 24.36) | 0.193(0.189,0.196) |
| Kingdom of the Netherlands | 0.67 (0.13, 1.28) | 0.60 (0.13, 1.11) | -0.003(-0.003,-0.002) | 17.96 (3.37, 33.79) | 14.90 (3.25, 27.37) | -0.107(-0.125,-0.090) |
| Kyrgyz Republic | 0.33 (0.07, 0.63) | 0.79 (0.19, 1.43) | 0.014(0.013,0.015) | 9.68 (2.18, 18.30) | 23.92 (5.78, 43.49) | 0.447(0.424,0.471) |
| Lao People's Democratic Republic | 0.03 (-0.01, 0.10) | 0.17 (0.02, 0.37) | 0.005(0.005,0.005) | 1.04 (-0.21, 3.39) | 5.82 (0.63, 12.61) | 0.156(0.155,0.158) |
| Lebanese Republic | 0.40 (0.08, 0.89) | 0.71 (0.17, 1.27) | 0.010(0.009,0.010) | 11.26 (2.33, 24.63) | 18.81 (4.70, 33.92) | 0.246(0.232,0.260) |
| Malaysia | 0.14 (0.02, 0.29) | 0.38 (0.09, 0.72) | 0.008(0.007,0.008) | 4.69 (0.85, 9.63) | 11.96 (2.89, 23.29) | 0.228(0.218,0.237) |
| Mongolia | 0.26 (0.04, 0.55) | 0.42 (0.08, 0.83) | 0.005(0.005,0.005) | 8.48 (1.32, 17.89) | 12.83 (2.61, 25.88) | 0.137(0.131,0.143) |
| Montenegro | 0.49 (0.11, 0.96) | 0.77 (0.20, 1.42) | 0.009(0.008,0.010) | 14.46 (3.27, 28.03) | 20.64 (5.39, 38.29) | 0.202(0.181,0.223) |
| New Zealand | 0.73 (0.16, 1.34) | 0.51 (0.12, 0.92) | -0.008(-0.008,-0.007) | 20.75 (4.70, 37.82) | 13.57 (3.25, 24.14) | -0.217(-0.258,-0.176) |
| North Macedonia | 0.58 (0.13, 1.14) | 0.93 (0.23, 1.74) | 0.011(0.011,0.011) | 16.79 (3.82, 32.74) | 25.07 (6.38, 47.79) | 0.274(0.260,0.288) |
| Northern Mariana Islands | 0.24 (0.05, 0.50) | 0.62 (0.16, 1.07) | 0.012(0.011,0.012) | 7.79 (1.80, 16.89) | 19.71 (5.46, 33.67) | 0.383(0.362,0.403) |
| Palestine | 0.32 (0.06, 0.69) | 0.64 (0.18, 1.18) | 0.010(0.010,0.011) | 9.25 (1.84, 20.48) | 18.12 (5.05, 33.49) | 0.280(0.274,0.286) |
| People's Democratic Republic of Algeria | 0.12 (0.02, 0.24) | 0.25 (0.07, 0.47) | 0.004(0.004,0.004) | 3.35 (0.68, 6.70) | 6.86 (1.81, 12.54) | 0.114(0.113,0.114) |
| People's Republic of Bangladesh | 0.01 (-0.01, 0.03) | 0.11 (0.01, 0.24) | 0.003(0.003,0.003) | 0.26 (-0.37, 1.12) | 3.65 (0.41, 8.22) | 0.111(0.110,0.112) |
| People's Republic of China | 0.03 (-0.02, 0.08) | 0.16 (0.03, 0.32) | 0.004(0.004,0.004) | 0.98 (-0.47, 2.67) | 4.85 (0.96, 9.92) | 0.126(0.125,0.127) |
| Plurinational State of Bolivia | 0.14 (0.02, 0.36) | 0.47 (0.12, 0.94) | 0.011(0.011,0.011) | 4.52 (0.60, 11.10) | 14.42 (3.54, 28.76) | 0.319(0.318,0.320) |
| Portuguese Republic | 0.33 (0.06, 0.63) | 0.39 (0.09, 0.71) | 0.003(0.002,0.003) | 9.25 (1.66, 18.10) | 10.34 (2.43, 18.64) | 0.055(0.036,0.074) |
| Principality of Andorra | 0.19 (0.03, 0.41) | 0.18 (0.04, 0.36) | -0.000(-0.001,-0.000) | 5.00 (0.82, 11.07) | 4.71 (1.02, 9.76) | -0.014(-0.021,-0.008) |
| Principality of Monaco | 0.61 (0.11, 1.25) | 0.67 (0.15, 1.45) | 0.002(0.002,0.002) | 17.17 (3.18, 35.59) | 17.88 (4.02, 38.56) | 0.025(0.019,0.031) |
| Puerto Rico | 0.27 (0.06, 0.48) | 0.59 (0.16, 1.05) | 0.010(0.010,0.011) | 7.58 (1.85, 13.29) | 17.59 (5.08, 31.02) | 0.324(0.304,0.344) |
| Republic of Albania | 0.19 (0.04, 0.37) | 0.29 (0.07, 0.57) | 0.003(0.003,0.003) | 5.17 (1.07, 10.40) | 7.78 (1.85, 15.22) | 0.079(0.071,0.087) |
| Republic of Angola | 0.03 (-0.00, 0.09) | 0.16 (0.02, 0.34) | 0.004(0.004,0.004) | 1.07 (-0.02, 2.78) | 5.02 (0.75, 10.83) | 0.126(0.125,0.128) |
| Republic of Armenia | 0.47 (0.11, 0.87) | 0.73 (0.20, 1.31) | 0.009(0.007,0.010) | 13.69 (3.21, 26.03) | 20.19 (5.51, 36.53) | 0.211(0.183,0.239) |
| Republic of Austria | 0.72 (0.14, 1.37) | 0.51 (0.11, 0.98) | -0.007(-0.008,-0.006) | 18.49 (3.53, 35.23) | 12.24 (2.65, 23.10) | -0.211(-0.235,-0.187) |
| Republic of Azerbaijan | 0.22 (0.04, 0.45) | 0.38 (0.09, 0.77) | 0.005(0.005,0.005) | 7.18 (1.47, 14.55) | 11.96 (2.79, 24.80) | 0.155(0.147,0.163) |
| Republic of Belarus | 0.49 (0.11, 0.93) | 0.77 (0.18, 1.45) | 0.009(0.007,0.012) | 14.74 (3.31, 27.93) | 22.80 (5.31, 42.73) | 0.198(0.131,0.264) |
| Republic of Benin | 0.10 (0.02, 0.20) | 0.17 (0.04, 0.32) | 0.002(0.002,0.002) | 3.11 (0.61, 6.15) | 5.08 (1.16, 10.09) | 0.064(0.061,0.066) |
| Republic of Botswana | 0.25 (0.05, 0.53) | 0.59 (0.14, 1.10) | 0.011(0.010,0.011) | 7.20 (1.59, 16.01) | 16.40 (3.78, 31.67) | 0.281(0.266,0.296) |
| Republic of Bulgaria | 0.58 (0.14, 1.05) | 0.98 (0.23, 1.86) | 0.014(0.013,0.016) | 17.71 (4.22, 31.77) | 29.08 (7.02, 55.46) | 0.413(0.368,0.457) |
| Republic of Burundi | 0.04 (-0.02, 0.12) | 0.11 (0.01, 0.25) | 0.002(0.002,0.002) | 1.27 (-0.46, 3.81) | 3.12 (0.18, 7.32) | 0.059(0.058,0.061) |
| Republic of Cabo Verde | 0.03 (0.01, 0.06) | 0.20 (0.04, 0.41) | 0.006(0.005,0.006) | 0.97 (0.18, 2.02) | 5.95 (1.33, 12.28) | 0.163(0.161,0.165) |
| Republic of Cameroon | 0.15 (0.03, 0.30) | 0.30 (0.07, 0.61) | 0.005(0.005,0.005) | 4.44 (0.94, 8.89) | 8.78 (2.01, 17.74) | 0.140(0.138,0.142) |
| Republic of Chad | 0.04 (0.01, 0.09) | 0.09 (0.01, 0.20) | 0.002(0.002,0.002) | 1.22 (0.18, 2.63) | 2.63 (0.41, 5.78) | 0.045(0.045,0.046) |
| Republic of Chile | 0.44 (0.10, 0.82) | 0.61 (0.16, 1.06) | 0.005(0.004,0.007) | 12.76 (2.82, 23.85) | 17.72 (4.65, 30.70) | 0.174(0.145,0.204) |
| Republic of Colombia | 0.33 (0.06, 0.64) | 0.59 (0.14, 1.07) | 0.009(0.008,0.009) | 9.63 (1.86, 18.75) | 17.70 (4.19, 32.81) | 0.271(0.261,0.280) |
| Republic of Costa Rica | 0.20 (0.04, 0.39) | 0.46 (0.11, 0.83) | 0.008(0.007,0.009) | 6.04 (1.32, 11.58) | 14.29 (3.53, 25.48) | 0.258(0.236,0.280) |
| Republic of Croatia | 0.71 (0.16, 1.34) | 0.90 (0.23, 1.61) | 0.006(0.004,0.007) | 19.49 (4.39, 35.96) | 24.10 (5.94, 43.40) | 0.128(0.085,0.172) |
| Republic of Cuba | 0.19 (0.04, 0.37) | 0.47 (0.11, 0.87) | 0.009(0.008,0.010) | 6.04 (1.30, 11.51) | 14.64 (3.53, 27.15) | 0.293(0.273,0.313) |
| Republic of Cyprus | 0.37 (0.06, 0.78) | 0.56 (0.12, 1.09) | 0.006(0.005,0.007) | 9.58 (1.71, 19.80) | 14.18 (3.16, 27.60) | 0.144(0.127,0.160) |
| Republic of Côte d'Ivoire | 0.15 (0.03, 0.29) | 0.31 (0.06, 0.61) | 0.005(0.005,0.005) | 4.58 (0.88, 8.98) | 9.22 (1.78, 18.67) | 0.152(0.147,0.156) |
| Republic of Djibouti | 0.03 (-0.02, 0.11) | 0.12 (-0.00, 0.30) | 0.003(0.003,0.003) | 1.14 (-0.61, 3.48) | 4.00 (0.14, 9.59) | 0.091(0.089,0.093) |
| Republic of Ecuador | 0.05 (0.01, 0.09) | 0.58 (0.14, 1.05) | 0.017(0.016,0.018) | 1.57 (0.32, 2.94) | 17.81 (4.36, 31.92) | 0.517(0.494,0.541) |
| Republic of El Salvador | 0.23 (0.05, 0.45) | 0.58 (0.15, 1.09) | 0.011(0.011,0.012) | 7.20 (1.61, 14.15) | 18.13 (4.79, 34.11) | 0.355(0.346,0.363) |
| Republic of Equatorial Guinea | 0.09 (0.01, 0.20) | 0.36 (0.07, 0.73) | 0.009(0.009,0.009) | 2.89 (0.38, 6.43) | 11.11 (2.13, 22.48) | 0.266(0.263,0.270) |
| Republic of Estonia | 0.83 (0.19, 1.54) | 0.79 (0.18, 1.42) | 0.001(-0.003,0.006) | 24.47 (5.71, 45.23) | 21.08 (4.91, 37.97) | -0.105(-0.234,0.024) |
| Republic of Fiji | 0.24 (0.06, 0.48) | 0.40 (0.12, 0.71) | 0.005(0.005,0.005) | 7.63 (1.82, 15.40) | 12.15 (3.65, 22.12) | 0.148(0.144,0.151) |
| Republic of Finland | 0.64 (0.12, 1.21) | 0.57 (0.13, 1.04) | -0.003(-0.003,-0.002) | 17.05 (3.29, 32.44) | 14.44 (3.33, 26.55) | -0.082(-0.114,-0.050) |
| Republic of Ghana | 0.05 (0.00, 0.10) | 0.22 (0.05, 0.45) | 0.006(0.006,0.006) | 1.66 (0.19, 3.44) | 6.96 (1.59, 14.12) | 0.174(0.172,0.176) |
| Republic of Guatemala | 0.11 (0.03, 0.20) | 0.31 (0.08, 0.57) | 0.006(0.006,0.007) | 3.34 (0.80, 6.21) | 9.62 (2.51, 17.82) | 0.197(0.186,0.209) |
| Republic of Guinea | 0.07 (0.01, 0.14) | 0.14 (0.03, 0.28) | 0.002(0.002,0.002) | 2.19 (0.37, 4.36) | 4.26 (0.90, 8.74) | 0.067(0.066,0.067) |
| Republic of Guinea-Bissau | 0.07 (0.01, 0.15) | 0.16 (0.03, 0.32) | 0.003(0.003,0.003) | 2.14 (0.30, 4.78) | 5.01 (0.87, 10.28) | 0.094(0.093,0.095) |
| Republic of Guyana | 0.35 (0.06, 0.69) | 0.80 (0.19, 1.49) | 0.015(0.013,0.016) | 11.50 (2.06, 22.39) | 25.72 (6.16, 47.45) | 0.460(0.415,0.505) |
| Republic of Haiti | 0.07 (-0.01, 0.22) | 0.23 (0.03, 0.54) | 0.005(0.005,0.005) | 2.53 (-0.09, 7.72) | 7.86 (0.92, 17.77) | 0.175(0.173,0.176) |
| Republic of Honduras | 0.28 (0.05, 0.56) | 0.68 (0.17, 1.38) | 0.013(0.013,0.013) | 8.73 (1.56, 17.55) | 21.07 (5.34, 43.32) | 0.399(0.392,0.406) |
| Republic of Iceland | 0.82 (0.17, 1.57) | 0.65 (0.15, 1.23) | -0.006(-0.007,-0.005) | 22.45 (4.78, 42.45) | 16.69 (3.98, 31.22) | -0.219(-0.242,-0.196) |
| Republic of India | 0.03 (-0.01, 0.06) | 0.14 (0.02, 0.27) | 0.004(0.004,0.004) | 0.85 (-0.14, 1.98) | 4.34 (0.73, 8.35) | 0.114(0.113,0.115) |
| Republic of Indonesia | 0.02 (-0.01, 0.07) | 0.16 (0.02, 0.35) | 0.005(0.004,0.005) | 0.95 (-0.36, 2.74) | 5.86 (0.88, 12.53) | 0.160(0.159,0.161) |
| Republic of Iraq | 0.27 (0.06, 0.60) | 0.47 (0.12, 0.89) | 0.006(0.006,0.006) | 8.46 (1.80, 18.47) | 14.22 (3.65, 27.48) | 0.184(0.182,0.186) |
| Republic of Italy | 0.39 (0.08, 0.74) | 0.49 (0.11, 0.92) | 0.003(0.003,0.004) | 10.57 (1.98, 20.37) | 12.26 (2.70, 23.33) | 0.056(0.041,0.071) |
| Republic of Kazakhstan | 0.48 (0.11, 0.88) | 0.70 (0.18, 1.25) | 0.006(0.005,0.007) | 14.71 (3.26, 26.73) | 21.26 (5.30, 38.01) | 0.192(0.166,0.218) |
| Republic of Kenya | 0.08 (0.01, 0.17) | 0.31 (0.07, 0.63) | 0.007(0.007,0.007) | 2.65 (0.27, 5.73) | 10.11 (2.32, 20.55) | 0.240(0.239,0.242) |
| Republic of Kiribati | 0.05 (0.01, 0.10) | 0.12 (0.03, 0.24) | 0.002(0.002,0.002) | 1.80 (0.40, 3.52) | 4.04 (1.11, 8.00) | 0.073(0.072,0.074) |
| Republic of Korea | 0.02 (-0.01, 0.06) | 0.12 (0.02, 0.23) | 0.003(0.003,0.003) | 0.56 (-0.49, 2.06) | 3.37 (0.42, 6.47) | 0.091(0.090,0.092) |
| Republic of Latvia | 0.76 (0.18, 1.40) | 1.18 (0.29, 2.20) | 0.014(0.012,0.016) | 22.86 (5.43, 41.65) | 33.93 (8.25, 64.75) | 0.371(0.302,0.441) |
| Republic of Liberia | 0.11 (0.02, 0.21) | 0.25 (0.06, 0.50) | 0.005(0.005,0.005) | 3.32 (0.58, 6.58) | 7.65 (1.74, 15.24) | 0.134(0.130,0.138) |
| Republic of Lithuania | 0.72 (0.17, 1.36) | 0.99 (0.24, 1.83) | 0.010(0.008,0.012) | 20.58 (4.73, 39.44) | 27.08 (6.53, 50.53) | 0.240(0.180,0.300) |
| Republic of Madagascar | 0.07 (-0.00, 0.15) | 0.19 (0.03, 0.42) | 0.004(0.004,0.004) | 1.87 (-0.05, 4.34) | 5.53 (0.89, 12.31) | 0.116(0.115,0.118) |
| Republic of Malawi | 0.02 (-0.02, 0.06) | 0.11 (0.01, 0.28) | 0.003(0.003,0.003) | 0.68 (-0.41, 2.07) | 4.20 (0.45, 9.71) | 0.113(0.112,0.114) |
| Republic of Maldives | 0.10 (0.01, 0.33) | 0.31 (0.07, 0.63) | 0.006(0.006,0.007) | 3.79 (0.32, 12.49) | 10.94 (2.48, 22.79) | 0.229(0.224,0.234) |
| Republic of Mali | 0.03 (0.00, 0.07) | 0.06 (0.01, 0.13) | 0.001(0.001,0.001) | 1.03 (0.12, 2.08) | 1.89 (0.32, 3.89) | 0.028(0.028,0.028) |
| Republic of Malta | 0.56 (0.10, 1.05) | 0.75 (0.18, 1.40) | 0.006(0.005,0.007) | 15.09 (2.70, 28.56) | 20.05 (4.95, 37.32) | 0.114(0.079,0.150) |
| Republic of Mauritius | 0.18 (0.03, 0.35) | 0.57 (0.13, 1.05) | 0.013(0.012,0.014) | 6.01 (1.04, 11.71) | 19.03 (4.40, 35.59) | 0.443(0.404,0.482) |
| Republic of Moldova | 0.66 (0.16, 1.20) | 0.63 (0.16, 1.09) | -0.003(-0.004,-0.001) | 20.27 (4.84, 36.59) | 18.87 (4.87, 32.61) | -0.081(-0.121,-0.042) |
| Republic of Mozambique | 0.07 (-0.00, 0.17) | 0.26 (0.04, 0.58) | 0.006(0.006,0.006) | 2.26 (-0.07, 5.30) | 8.52 (1.39, 18.55) | 0.202(0.200,0.203) |
| Republic of Namibia | 0.15 (0.03, 0.29) | 0.38 (0.09, 0.77) | 0.008(0.008,0.008) | 4.45 (0.90, 8.76) | 10.98 (2.44, 22.06) | 0.211(0.209,0.213) |
| Republic of Nauru | 0.29 (0.07, 0.66) | 0.55 (0.12, 1.19) | 0.008(0.008,0.008) | 10.00 (2.46, 21.93) | 18.25 (4.11, 40.30) | 0.266(0.261,0.270) |
| Republic of Nicaragua | 0.16 (0.03, 0.32) | 0.37 (0.10, 0.70) | 0.007(0.006,0.007) | 4.86 (1.10, 10.02) | 11.25 (3.07, 21.36) | 0.202(0.195,0.209) |
| Republic of Niue | 0.22 (0.05, 0.43) | 0.50 (0.14, 0.97) | 0.009(0.009,0.009) | 7.30 (1.69, 14.31) | 16.55 (4.73, 31.87) | 0.305(0.296,0.314) |
| Republic of Palau | 0.07 (0.01, 0.13) | 0.11 (0.03, 0.20) | 0.001(0.001,0.002) | 2.02 (0.46, 3.86) | 3.24 (0.90, 6.05) | 0.039(0.039,0.040) |
| Republic of Panama | 0.18 (0.04, 0.33) | 0.53 (0.16, 0.93) | 0.012(0.011,0.012) | 4.68 (1.01, 8.71) | 15.35 (4.40, 27.19) | 0.354(0.325,0.382) |
| Republic of Paraguay | 0.16 (0.03, 0.35) | 0.38 (0.08, 0.75) | 0.007(0.007,0.007) | 4.91 (0.94, 10.42) | 11.25 (2.55, 21.94) | 0.204(0.199,0.209) |
| Republic of Peru | 0.22 (0.04, 0.45) | 0.47 (0.11, 0.93) | 0.008(0.008,0.009) | 7.00 (1.25, 14.29) | 14.78 (3.48, 29.47) | 0.251(0.237,0.264) |
| Republic of Poland | 0.85 (0.19, 1.55) | 1.08 (0.28, 1.98) | 0.007(0.006,0.007) | 24.84 (5.31, 45.09) | 28.45 (7.06, 52.51) | 0.098(0.068,0.127) |
| Republic of Rwanda | 0.06 (-0.02, 0.18) | 0.23 (0.03, 0.53) | 0.005(0.005,0.005) | 1.99 (-0.56, 5.70) | 7.19 (0.88, 16.41) | 0.166(0.162,0.170) |
| Republic of San Marino | 0.38 (0.08, 0.76) | 0.27 (0.06, 0.57) | -0.004(-0.004,-0.003) | 9.74 (2.08, 19.11) | 7.23 (1.46, 15.14) | -0.095(-0.109,-0.081) |
| Republic of Senegal | 0.08 (0.01, 0.15) | 0.18 (0.04, 0.37) | 0.003(0.003,0.003) | 2.48 (0.42, 4.92) | 5.65 (1.14, 11.54) | 0.103(0.097,0.110) |
| Republic of Serbia | 0.61 (0.13, 1.18) | 1.08 (0.33, 1.92) | 0.015(0.014,0.015) | 17.63 (3.84, 34.01) | 30.12 (8.99, 53.93) | 0.404(0.387,0.421) |
| Republic of Seychelles | 0.46 (0.10, 0.89) | 1.03 (0.28, 1.82) | 0.018(0.017,0.019) | 16.17 (3.51, 30.83) | 34.30 (9.50, 60.74) | 0.585(0.573,0.597) |
| Republic of Sierra Leone | 0.05 (0.01, 0.11) | 0.13 (0.02, 0.27) | 0.003(0.002,0.003) | 1.56 (0.19, 3.29) | 3.91 (0.70, 8.25) | 0.076(0.075,0.077) |
| Republic of Singapore | 0.06 (-0.03, 0.17) | 0.25 (0.06, 0.46) | 0.006(0.006,0.006) | 2.21 (-0.69, 5.76) | 7.64 (1.76, 13.89) | 0.178(0.167,0.189) |
| Republic of Slovenia | 0.74 (0.17, 1.32) | 0.71 (0.18, 1.30) | -0.001(-0.003,-0.000) | 20.77 (4.81, 36.73) | 18.27 (4.56, 33.97) | -0.110(-0.168,-0.051) |
| Republic of South Africa | 0.39 (0.10, 0.74) | 0.83 (0.23, 1.47) | 0.014(0.013,0.014) | 11.67 (3.04, 21.90) | 23.60 (6.60, 41.60) | 0.372(0.362,0.383) |
| Republic of South Sudan | 0.03 (-0.02, 0.09) | 0.06 (-0.02, 0.17) | 0.001(0.001,0.001) | 1.00 (-0.50, 2.91) | 2.03 (-0.44, 5.47) | 0.033(0.032,0.033) |
| Republic of Sudan | 0.12 (0.02, 0.33) | 0.27 (0.07, 0.58) | 0.005(0.005,0.005) | 3.54 (0.52, 10.02) | 8.27 (2.04, 17.55) | 0.152(0.151,0.153) |
| Republic of Suriname | 0.24 (0.03, 0.51) | 0.49 (0.11, 0.96) | 0.008(0.008,0.009) | 7.59 (1.08, 15.75) | 15.79 (3.57, 30.18) | 0.263(0.251,0.275) |
| Republic of Tajikistan | 0.15 (0.03, 0.31) | 0.20 (0.05, 0.40) | 0.001(0.001,0.002) | 4.58 (0.97, 9.23) | 6.20 (1.41, 12.51) | 0.050(0.047,0.054) |
| Republic of Trinidad and Tobago | 0.64 (0.15, 1.22) | 0.92 (0.25, 1.66) | 0.009(0.008,0.010) | 20.53 (4.79, 38.00) | 29.37 (8.20, 54.33) | 0.301(0.267,0.334) |
| Republic of Tunisia | 0.19 (0.04, 0.37) | 0.45 (0.12, 0.88) | 0.008(0.008,0.008) | 5.69 (1.23, 10.86) | 13.27 (3.54, 25.30) | 0.242(0.241,0.244) |
| Republic of Turkey | 0.54 (0.12, 1.12) | 0.77 (0.22, 1.40) | 0.007(0.007,0.008) | 16.17 (3.46, 33.96) | 22.07 (6.07, 39.86) | 0.180(0.174,0.187) |
| Republic of Uganda | 0.08 (-0.01, 0.21) | 0.31 (0.04, 0.65) | 0.007(0.007,0.007) | 2.71 (-0.19, 6.40) | 9.97 (1.34, 21.02) | 0.235(0.233,0.238) |
| Republic of Uzbekistan | 0.17 (0.04, 0.33) | 0.35 (0.08, 0.68) | 0.007(0.006,0.007) | 5.31 (1.12, 10.49) | 11.07 (2.53, 21.64) | 0.178(0.168,0.187) |
| Republic of Vanuatu | 0.06 (0.01, 0.15) | 0.16 (0.03, 0.37) | 0.003(0.003,0.003) | 1.98 (0.28, 5.11) | 5.03 (0.98, 12.10) | 0.097(0.095,0.099) |
| Republic of Yemen | 0.04 (0.00, 0.13) | 0.13 (0.02, 0.29) | 0.003(0.003,0.003) | 1.38 (0.13, 3.94) | 3.88 (0.72, 8.52) | 0.082(0.080,0.083) |
| Republic of Zambia | 0.09 (-0.01, 0.22) | 0.44 (0.10, 0.89) | 0.011(0.011,0.011) | 2.81 (-0.14, 6.72) | 13.44 (2.93, 26.94) | 0.343(0.341,0.346) |
| Republic of Zimbabwe | 0.20 (0.04, 0.42) | 0.77 (0.18, 1.49) | 0.018(0.018,0.019) | 6.37 (1.25, 13.11) | 23.47 (5.52, 46.01) | 0.551(0.543,0.558) |
| Republic of the Congo | 0.10 (0.01, 0.23) | 0.38 (0.08, 0.74) | 0.009(0.009,0.009) | 3.17 (0.27, 7.56) | 11.82 (2.47, 22.94) | 0.282(0.278,0.286) |
| Republic of the Gambia | 0.08 (0.01, 0.16) | 0.18 (0.04, 0.38) | 0.003(0.003,0.003) | 2.56 (0.46, 5.00) | 5.57 (1.23, 11.92) | 0.097(0.090,0.104) |
| Republic of the Marshall Islands | 0.22 (0.05, 0.47) | 0.50 (0.11, 1.07) | 0.009(0.009,0.009) | 7.14 (1.81, 14.85) | 15.97 (3.58, 35.65) | 0.287(0.284,0.290) |
| Republic of the Niger | 0.04 (0.01, 0.09) | 0.08 (0.01, 0.17) | 0.001(0.001,0.001) | 1.41 (0.17, 2.99) | 2.34 (0.40, 5.35) | 0.031(0.030,0.031) |
| Republic of the Philippines | 0.10 (0.00, 0.20) | 0.30 (0.05, 0.56) | 0.006(0.006,0.007) | 3.32 (0.23, 6.87) | 10.13 (1.75, 19.50) | 0.219(0.217,0.221) |
| Republic of the Union of Myanmar | 0.06 (-0.00, 0.16) | 0.16 (0.02, 0.34) | 0.003(0.003,0.003) | 2.38 (0.05, 5.77) | 5.70 (0.76, 11.91) | 0.107(0.107,0.108) |
| Romania | 0.48 (0.10, 0.87) | 0.80 (0.18, 1.48) | 0.010(0.010,0.011) | 15.18 (3.32, 28.39) | 22.96 (5.19, 42.09) | 0.248(0.229,0.266) |
| Russian Federation | 0.75 (0.18, 1.35) | 0.94 (0.25, 1.64) | 0.005(0.004,0.007) | 23.50 (5.58, 42.28) | 27.59 (7.42, 47.69) | 0.062(0.018,0.105) |
| Saint Kitts and Nevis | 0.51 (0.10, 1.07) | 0.80 (0.21, 1.45) | 0.011(0.010,0.012) | 15.96 (3.21, 34.22) | 22.27 (6.25, 40.27) | 0.230(0.206,0.254) |
| Saint Lucia | 0.43 (0.08, 0.86) | 0.75 (0.20, 1.41) | 0.010(0.009,0.011) | 14.94 (3.34, 29.57) | 24.42 (6.82, 44.54) | 0.311(0.289,0.332) |
| Saint Vincent and the Grenadines | 0.28 (0.04, 0.57) | 0.59 (0.14, 1.12) | 0.010(0.010,0.010) | 9.04 (1.44, 18.37) | 19.45 (4.68, 36.54) | 0.330(0.320,0.341) |
| Slovak Republic | 0.90 (0.22, 1.71) | 0.97 (0.27, 1.76) | 0.002(0.002,0.003) | 25.98 (6.12, 49.55) | 26.57 (7.38, 47.68) | 0.030(0.011,0.049) |
| Socialist Republic of Viet Nam | 0.00 (-0.01, 0.01) | 0.05 (-0.00, 0.11) | 0.001(0.001,0.001) | 0.03 (-0.30, 0.40) | 1.45 (0.02, 3.43) | 0.046(0.046,0.047) |
| Solomon Islands | 0.08 (0.01, 0.19) | 0.21 (0.04, 0.47) | 0.004(0.004,0.004) | 2.77 (0.43, 6.83) | 7.51 (1.57, 16.99) | 0.156(0.152,0.160) |
| State of Eritrea | 0.04 (-0.00, 0.10) | 0.13 (0.01, 0.29) | 0.003(0.003,0.003) | 1.30 (-0.10, 3.23) | 4.06 (0.29, 8.86) | 0.089(0.087,0.090) |
| State of Israel | 0.72 (0.15, 1.36) | 0.58 (0.14, 1.04) | -0.005(-0.006,-0.003) | 19.33 (4.21, 36.73) | 14.50 (3.38, 26.09) | -0.170(-0.202,-0.137) |
| State of Kuwait | 0.52 (0.14, 0.93) | 0.55 (0.17, 0.94) | 0.000(-0.001,0.001) | 15.04 (4.02, 26.71) | 15.58 (4.86, 26.41) | 0.003(-0.041,0.047) |
| State of Libya | 0.39 (0.08, 0.80) | 1.06 (0.28, 1.96) | 0.021(0.021,0.022) | 11.66 (2.36, 24.21) | 30.83 (8.32, 57.05) | 0.618(0.612,0.623) |
| State of Qatar | 0.67 (0.15, 1.36) | 1.23 (0.38, 2.27) | 0.018(0.016,0.020) | 18.63 (4.15, 37.22) | 31.93 (9.75, 58.89) | 0.395(0.348,0.443) |
| Sultanate of Oman | 0.11 (0.02, 0.24) | 0.29 (0.08, 0.51) | 0.006(0.005,0.006) | 3.48 (0.57, 7.61) | 8.44 (2.43, 15.01) | 0.163(0.155,0.172) |
| Swiss Confederation | 0.32 (0.06, 0.59) | 0.37 (0.08, 0.69) | 0.001(0.000,0.002) | 8.48 (1.53, 15.87) | 8.54 (1.77, 15.89) | 0.017(-0.012,0.047) |
| Syrian Arab Republic | 0.16 (0.03, 0.34) | 0.38 (0.11, 0.69) | 0.007(0.007,0.007) | 4.97 (1.01, 10.05) | 10.90 (3.14, 19.84) | 0.192(0.189,0.195) |
| Taiwan (Province of China) | 0.07 (0.00, 0.14) | 0.26 (0.05, 0.49) | 0.006(0.006,0.007) | 1.89 (-0.05, 4.03) | 8.51 (1.65, 15.85) | 0.229(0.212,0.246) |
| Togolese Republic | 0.07 (0.01, 0.14) | 0.17 (0.03, 0.37) | 0.003(0.003,0.003) | 2.14 (0.37, 4.52) | 5.33 (1.07, 10.96) | 0.102(0.101,0.103) |
| Tokelau | 0.18 (0.04, 0.39) | 0.42 (0.12, 0.83) | 0.008(0.007,0.008) | 6.29 (1.36, 13.14) | 14.17 (4.22, 27.87) | 0.260(0.252,0.268) |
| Turkmenistan | 0.19 (0.04, 0.36) | 0.42 (0.09, 0.82) | 0.008(0.007,0.009) | 5.97 (1.20, 11.27) | 13.47 (2.79, 26.25) | 0.122(0.029,0.216) |
| Tuvalu | 0.14 (0.03, 0.30) | 0.38 (0.10, 0.74) | 0.008(0.008,0.008) | 4.77 (0.95, 10.10) | 12.34 (3.24, 24.38) | 0.246(0.243,0.249) |
| Ukraine | 0.54 (0.12, 0.95) | 0.77 (0.18, 1.52) | 0.008(0.007,0.009) | 16.91 (3.82, 30.41) | 24.10 (5.51, 48.03) | 0.239(0.216,0.261) |
| Union of the Comoros | 0.09 (-0.00, 0.23) | 0.36 (0.06, 0.78) | 0.008(0.008,0.009) | 2.88 (-0.09, 6.96) | 11.05 (1.83, 23.83) | 0.263(0.254,0.272) |
| United Arab Emirates | 0.83 (0.14, 2.00) | 3.73 (1.05, 6.55) | 0.094(0.088,0.101) | 24.32 (4.07, 60.28) | 80.08 (22.77, 140.04) | 1.811(1.694,1.928) |
| United Kingdom of Great Britain and Northern Ireland | 0.95 (0.21, 1.81) | 0.80 (0.20, 1.47) | -0.004(-0.005,-0.003) | 26.74 (5.93, 51.27) | 20.04 (4.89, 36.73) | -0.200(-0.217,-0.182) |
| United Mexican States | 0.40 (0.09, 0.73) | 0.77 (0.22, 1.41) | 0.012(0.012,0.013) | 11.95 (2.70, 21.86) | 24.53 (6.95, 44.33) | 0.405(0.392,0.418) |
| United Republic of Tanzania | 0.18 (0.02, 0.35) | 0.52 (0.11, 1.00) | 0.011(0.011,0.011) | 5.51 (0.81, 10.77) | 15.58 (3.46, 30.62) | 0.323(0.320,0.325) |
| United States Virgin Islands | 0.71 (0.17, 1.35) | 0.81 (0.23, 1.56) | 0.003(0.001,0.005) | 21.12 (5.28, 39.81) | 25.80 (7.26, 49.39) | 0.150(0.095,0.205) |
| United States of America | 0.86 (0.21, 1.55) | 0.79 (0.22, 1.37) | -0.002(-0.003,-0.002) | 24.00 (5.91, 43.36) | 20.97 (5.83, 36.19) | -0.105(-0.121,-0.089) |

Table S4 The ASMR, ASDR and their AAPC of ovarian cancer attributable to high body-mass index in 5 SDI and 21 GBD regions in 1990 and 2021.

| Location | Death | DALYs |
| --- | --- | --- |
| Global | 9.32(2.25, 16.62) | 6.74(1.31, 13.02) |
| High SDI | 11.2(2.75, 20.03) | 4.29(0.71, 8.14) |
| High-middle SDI | 11.48(2.77, 20.56) | 6.24(1.35, 11.33) |
| Middle SDI | 8.31(1.96, 15.16) | 8.45(2, 15.31) |
| Low-middle SDI | 6.01(1.28, 10.93) | 11.32(2.71, 20.37) |
| Low SDI | 3.94(0.64, 7.48) | 11.32(2.84, 20.18) |
| East Asia | 6.73(1.32, 13) | 10.09(2.51, 18.19) |
| Oceania | 9.43(2.25, 17.09) | 9.18(2.23, 16.35) |
| Southeast Asia | 5.07(0.9, 9.45) | 12.51(3.17, 22.52) |
| Central Asia | 12.91(3.29, 23.05) | 12.69(3.21, 22.68) |
| High-income Asia Pacific | 3.64(0.36, 7.13) | 5.38(1.03, 9.95) |
| Eastern Europe | 14.89(4.1, 25.8) | 3.54(0.37, 7.04) |
| Central Europe | 12.75(3.23, 22.88) | 14.64(4.01, 25.21) |
| Western Europe | 10.46(2.39, 19.29) | 14.19(3.74, 24.89) |
| Australasia | 13.15(3.42, 22.86) | 13.44(3.51, 23.48) |
| Southern Latin America | 14.12(3.7, 24.93) | 10.5(2.42, 19.28) |
| High-income North America | 14.26(3.82, 24.75) | 11.42(3.13, 20.07) |
| Andean Latin America | 11.89(2.92, 21.4) | 14.71(4.05, 25.3) |
| Caribbean | 11.05(2.96, 19.43) | 16.62(4.66, 28.14) |
| Central Latin America | 13.62(3.53, 24.17) | 12.4(3.15, 22.27) |
| South Asia | 4.15(0.69, 7.71) | 13.83(3.63, 24.43) |
| Tropical Latin America | 11.47(2.69, 21.14) | 11.52(2.76, 20.97) |
| Southern Sub-Saharan Africa | 15.08(4.23, 25.88) | 6.14(1.19, 10.83) |
| Central Sub-Saharan Africa | 5.92(1.11, 10.67) | 8.43(1.9, 15.45) |
| North Africa and Middle East | 16.3(4.53, 27.77) | 4.41(0.74, 8.11) |
| Eastern Sub-Saharan Africa | 4.8(0.83, 9.08) | 5.18(0.94, 9.87) |
| Western Sub-Saharan Africa | 8.25(1.85, 15.11) | 15.44(4.38, 26.63) |
